# Pyridinic-N Coordination Effect on the Adsorption and Activation of CO_2_ by Single Vacancy Iron-Doped Graphene

**DOI:** 10.1021/acs.langmuir.3c03327

**Published:** 2024-03-18

**Authors:** Hugo Cabrera-Tinoco, Luis Borja-Castro, Renato Valencia-Bedregal, Adela Perez-Carreño, Aldo Lalupu-García, Ismael Veliz-Quiñones, Angel Guillermo Bustamante Dominguez, Crispin H. W. Barnes, Luis De Los Santos Valladares

**Affiliations:** † Área de Ciencias Básicas, Universidad Continental, Lima 15311, Peru; ‡ Laboratorio de Cerámicos y Nanomateriales, Facultad de Ciencias Físicas, Universidad Nacional Mayor de San Marcos, Ap. Postal 14-0149 Lima, Peru; § Programa de Pós-Graduação em Ciências de Materiais, Centro de Ciências Exatas e da Natureza, Universidade Federal de Pernambuco, 50670-901 Recife, PE, Brazil; ∥ Cavendish Laboratory, Department of Physics, University of Cambridge, J. J Thomson Av, Cambridge CB3 0H3, U.K.

## Abstract

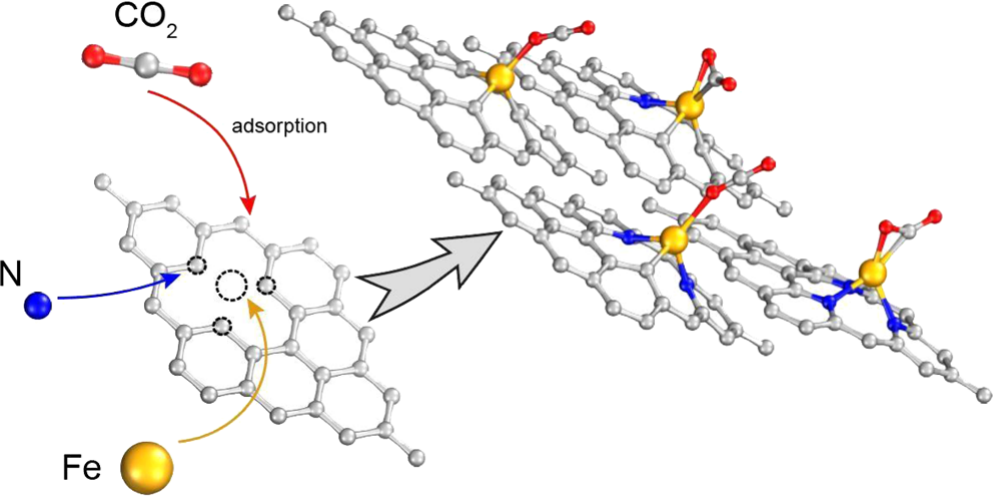

Graphene doped with different transition metals has been recently proposed to adsorb CO_2_ and help reduce the greenhouse effect. Iron-doped graphene is one of the most promising candidates for this task, but there is still a lack of full understanding of the adsorption mechanism. In this work, we analyze the electronic structure, geometry, and charge redistribution during adsorption of CO_2_ molecules by single vacancy iron-doped graphene by DFT calculations using the general gradient approximation of Perdew, Burke, and Ernzernhof functional (PBE) and the van der Waals density functional (vdW). To understand the impact of the pyridinic-N coordination of the iron atom, we gradually replaced the neighboring carbon atoms by nitrogen atoms. The analysis indicates that chemisorption and physisorption occur when the molecule is adsorbed in the side-on and end-on orientation, respectively. Adsorption is stronger when pyridinic-N coordination increases, and the vdW functional describes the chemical interactions and adsorption energy differently in relation to PBE without significant structural changes. The development of the chemical interactions with the change of coordination in the system is further investigated in this work with crystal overlap Hamilton population (COHP) analysis.

## Introduction

Carbon dioxide (CO_2_) is a harmful uncolored gas under standard conditions of pressure and temperature, and it is one of the main gases responsible for the greenhouse effect. Therefore, preventing its emission or searching for new technologies for its control have become important tasks to face in the coming decades.^1^ Some solid adsorbents, such as layered double hydroxides, zeolites, activated carbon, and graphene-based materials, have been proposed as good candidates to capture CO_2_ from flowing gases.^2^ In this way, carbonaceous solids could remain stable in humid conditions to be posteriorly regenerated and reused.^3^

In fact, graphene-based nanocomposites can adsorb molecules in the gas phase such as CO_2_.^4^ Since experimentally synthesized in 2004,^5^ graphene has been widely studied due to its mechanical, thermal, and electronic properties.^6,7^ However, it is almost chemically inert in its pure form, and it has a weak adsorption capacity basically due to van der Waals-type interactions^8^ Therefore, its potential use as an adsorbent agent is subjected to structural modifications.

It has been demonstrated that generating defects in the graphene structure substantially changes its properties.^9^ Doping graphene with different transition metals increases chemically active areas on its surface, thus making the material to become efficient in adsorbing gaseous molecules^10^ For example, techniques such as scanning transmission electron microscopy (STEM) can be used to dope graphene with transition metals in a controlled manner that just a single atom can be successfully replaced.^11^

Doping with iron and nitrogen atoms has been reported to improve considerably the adsorption properties,^12,13^ forming pyridinic-N bonds with two N–C or Fe–C bonds in a hexagon and being tested as sensors or as catalysts for heterogeneous reactions.^14^ For example, Dong et al. have recently inspected the simultaneous oxidation of NO and Hg^0^ to NO_2_ and HgO, respectively, by catalysis using Fe pyridinic-N4-doped graphene.^15^ Moreover, Nosheen et al. have also demonstrated that the same materials are effective for the chemisorption of CO, NO, and NO_2_ and the physisorption of CO_2_, H_2_S, and NH_3_.^16^

However, the adsorption of CO_2_ is weak for the case of iron-doped graphene, and there is some controversy regarding the chemical interaction between the molecule and the substrate. Cortés-Arriagada^17^ and Niwat Promthong^18^ have reported low adsorption energies such as −0.54 and −0.57 eV, respectively, but differing in the geometry of the adsorbed molecule. While Cortés-Arriagada proposes a bent geometry with an increase in the length of the bond closest to the iron atom, Promthong claims a linear geometry, vertically to the plane of graphene (end-on configuration). Besides, in a recent study, Nosheen et al. have reported an adsorption energy of −0.154 eV and geometries that are linear and parallel to the plane of the graphene.^18^ Further works about the chemisorption of graphene doped with other transition metal atoms such as Cu and Ni suggest that the structure of the molecule is not altered.^19^

The simultaneous doping of graphene with nitrogen and other transition metals has been claimed to have interesting catalytic properties. Recently, Chen et al. have studied the effect of the coordination environment on the electronic and reactivity properties of graphene codoped with iron, nitrogen, and boron related to the oxidation of NO and CO.^20^ Their results show that the catalytic property could be regulated by adjusting the coordination, thus suggesting that changes in coordination could affect other characteristics such as adsorption. The effect of pyridinic-N coordination on the adsorption of CO_2_ by graphene codoped with Fe and N and the lack of consensus about their chemical interaction and geometry motivate the present work.

In this article, the adsorption of CO_2_ by single vacancy iron-doped graphene was studied using density functional theory (DFT) calculations with two approaches (PBE and vdW). To understand the impact of the pyridinic-N coordination of the iron atom, the neighboring carbon atoms were gradually replaced by nitrogen atoms. In this way, the adsorption of CO_2_ on four different substrates with zero, one, two, and three nitrogen atoms in their structure was investigated. Initially, the structure of the system formed by the molecule adsorbed by the substrate was optimized looking for the lowest-energy configuration. Then, the stability of the system was analyzed through the adsorption energy, geometric parameters, and electronic structure by using the projected density of states (PDOS), charge density difference (CDD), and Bader population analysis. The chemical analysis of the interaction between the adsorbent and adsorbate was done by Crystal Orbital Hamilton Population (COHP). All of these features contribute to understanding the adsorption process in these systems.

## Computational Details

In this work, the SIESTA package^21^ has been used for the DFT calculations considering spin polarization. The interaction between nuclei and valence electrons was modeled by the norm-conserving pseudopotentials with the generalized gradient approximation of Perdew, Burke, and Ernzernhof (PBE).^22^ We also use the van der Waals density functional (vdW) to include the dispersion forces through the inclusion of nonlocal correlation.^2,3,23^ The orbitals were represented by a Linear Combination of Atomic Orbitals (LCAO) and expanded using a double-ζ plus polarization functions as a basis set (DZP). Because we have used an LCAO-type basis set, known as the basis set superposition, some error (BSSE) may occur. Eventual error would consist of the system converging to an artificial electronic configuration due to the imposition generated by the atomic orbitals. In the case of simulating an adsorption process, the erroneous superposition of the atomic orbitals of the adsorbent and adsorbate results in an overestimation of the interaction that manifests itself in a higher adsorption energy. To check the reliability of our results, we performed the counterpoise correction (CP).^24^ The methodology used is described in detail in Section S1.1, where Table S1 shows the contribution to the energy of each system considered for the CP correction of the CO_2_(e)@Fe-0N and CO_2_(s)@Fe-3N configurations. The results show that the correction by BSSE is less than a fraction of an eV (<0.2 eV), which indicates that the basis set used is reasonably adequate.^25−27^

The configuration of the pristine graphene was established as a 4 × 4 supercell (32 atoms). A carbon atom in the slab was replaced by an iron atom (Fe-0N) and then its coordination was modified by substituting the neighboring carbon atoms with one (Fe-1N), two (Fe-2N), and three nitrogen atoms (Fe-3N). A cell size validation test was performed. Adsorption calculations of the CO_2_ molecule in the end-on configuration were made using a 5 × 5 supercell (50 atoms) and all of the pyridinic coordinations. The binding energies, charge transference to the iron atom, and structural characteristics were compared and are provided in Table S2. Note that there is almost no difference between the results for both supercells. It should be noted that the supercell size used in this work is also used to study chemical processes or the effect of vacancies on graphene doped with transition metals.^28−31^ We also perform calculations with the GGA+*U* approximation^32^ to test the implication of the interaction between the d electrons of the Fe atom in the substrates. It was found that the use of this method does not contribute significantly to the results obtained in this work. This issue is discussed in some detail at the end of the section Geometric and Electronic Properties of the Substrates.

The Conjugate Gradient algorithm (CG) was used for the optimization of the structures. To avoid interaction between slabs, an empty space of 15 Å was left in the *z* direction. Structural relaxation was performed until the forces between the atoms and the variation in the total energy were less than 0.01 eV/Å and 1 × 10^–5^ eV, respectively. A 6 × 6 *k*-point grid (Monkhorst–Pack scheme) was used for the sampling of the Brillouin zone and 300 Ry mesh cutoff for geometry relaxation. To determine the electronic structure, a 7 × 7 *k*-point grid and a 350 Ry mesh cutoff were employed.

The characterization of the electronic structure was carried out using Projected Density of States (PDOS) and the chemical analysis by means of the Crystal Hamilton Orbital Population (COHP) method.^33^ It is important to mention that positive (negative) values of −COHP indicate bonding (antibonding) states, while for −COHP, values close to zero indicate nonbonding states. The integration of the COHP (ICOHP) up to the Fermi energy is a measure of the strength of the bond; i.e., the more negative the ICOHP is, the stronger the bond is. The Bader charge analysis was applied to determine the loss or gain of charge of the atoms of interest^34^. Spin polarization was considered for the PDOS, COHP (ICOHP), and Bader charge analysis in the study of all of the systems even for the nonmagnetic ones. The binding energy (*E*_bin_) of the iron atom on the single vacancy graphene is calculated by the following equation:
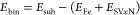
1where *E*_sub_, *E*_Fe_, and *E*_SVxN_ are the total energies of the substrate (Fe-1N, Fe-2N, or Fe-3N), free iron atom, and single vacancy graphene doped with *x* (*x* = 0,1,2 and 3) nitrogen atoms, respectively. The adsorption energy of the CO_2_ molecule (*E*_ads_) was calculated by
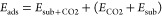
2where *E*_sub+CO2_, *E*_CO2_, and *E*_sub_ are the total energies of the system formed by the CO_2_ molecule adsorbed on the substrate, the isolated CO_2_ molecule, and the substrate defined above in eq 1, respectively. The negative value of *E*_bin_ [*E*_ads_] indicates that the bonding [adsorption] is exothermic, and when the value is larger, the bonding [adsorption] is more stable.

## Results and Discussion

### Geometric and Electronic Properties of the Substrates

**Figure 1:**
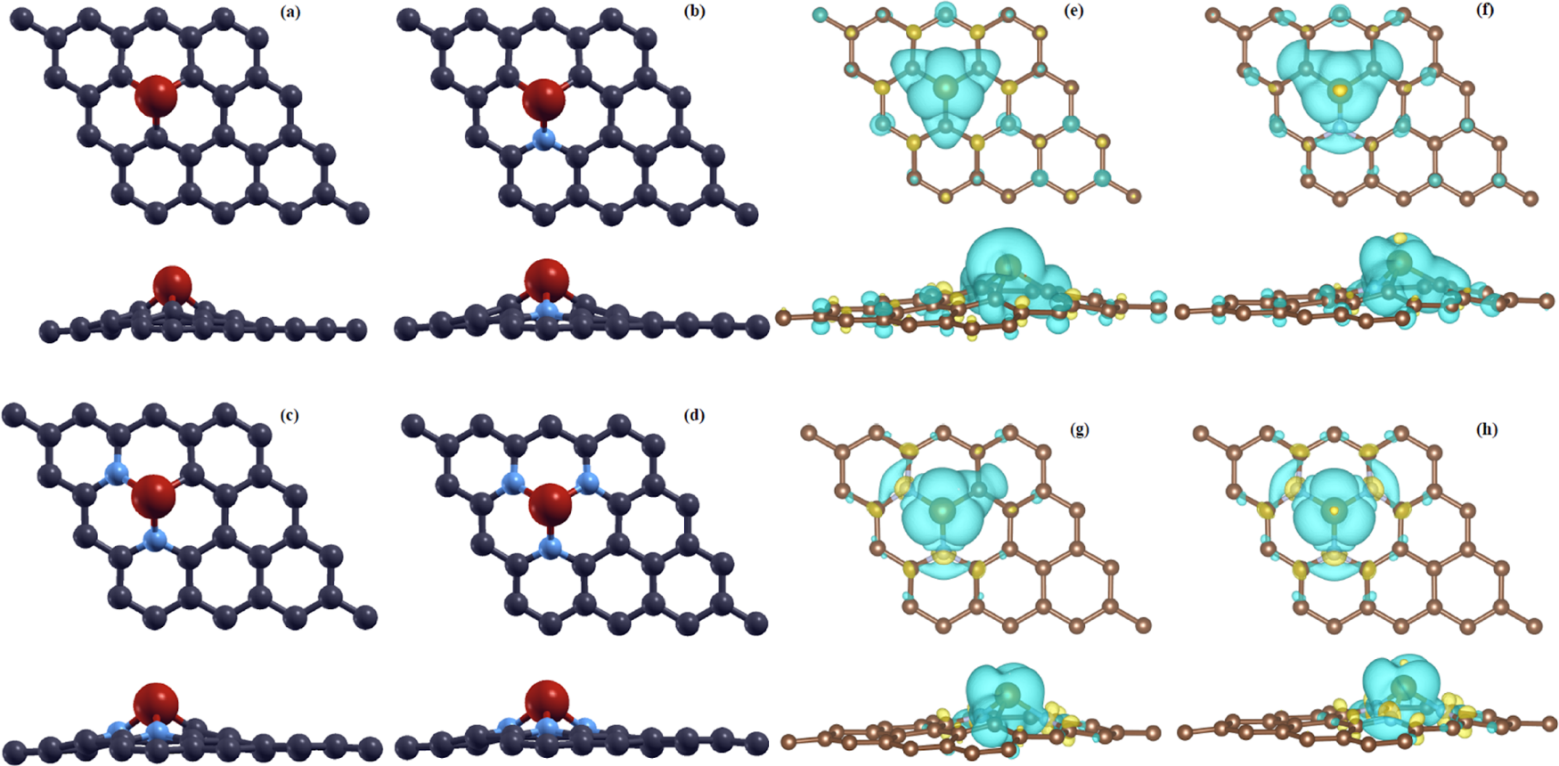
Relaxed structures for the Fe-0N (a), Fe-1N (b), Fe-2N (c), and Fe-3N (d) substrates. Charge density difference for Fe-0N (e), Fe-1N (f), Fe-2N (g), and Fe-3N (h) substrates (isosurface ≈0.003 e/bohr^3^).

The relaxed structures of the substrates Fe-0N, Fe-1N, Fe-2N, and Fe-3N are shown in Figure 1. The binding energy (*E*_bin_) (see Table 1), structural characteristics, and magnetic moment for the four substrates are listed in Table S3. According to the calculations, *E*_bin_ for the Fe-0N system is the lowest (−5.85 eV) and it decreases with coordination reaching the highest adsorption energy −3.02 eV for Fe-3N. It is clearly noted that when the number of nitrogen atoms in the substrate increases, the binding energy increases. The fact that the strongest and weakest bonds occur in Fe-0N and Fe-3N, respectively, has been reported in previous works. Chen et al. showed −7.46 and −4.31 eV for Fe-0N and Fe-3N, respectively.^20^ A similar trend is presented in the work of Gao et al., the binding energies are −7.14 and −4.41 eV for Fe-0N and Fe-3N, respectively.^35^ Yan et al. showed that the binding energies for Fe-0N and Fe-3N are −7.15 and −4.41 eV, respectively.^36^ The three mentioned works were carried out with the PBE functional. Yan et al. also reported the binding energy of Fe-0N and Fe-3N as −7.11 and −4.48 eV, respectively, using the empirical DFT-D3 functional to take account of dispersion effects.^14^ The works mentioned above were carried out using plane-wave basis set codes.

**Table 1:** Values of Binding energy (*E*_bin_) for Substrates and Adsorption Energy (*E*_ads_) for Adsorbate Systems

substrate	*E*_bin_ (eV)	adsorption system	*E*_ads_ (eV)
Fe-0N	–5.847	CO_2_(*e*)@Fe-0N	–0.412
		CO_2_(*s*)@Fe-0N	–0.517
Fe-1N	–4.033	CO_2_(*e*)@Fe-1N	–0.682
		CO_2_(*s*)@Fe-1N	–0.926
Fe-2N	–4.104	CO_2_(*e*)@Fe-2N	–1.353
		CO_2_(*s*)@Fe-2N	–1.792
Fe-3N	–3.023	CO_2_(*e*)@Fe-3N	–1.962
		CO_2_(*s*)@Fe-3N	–2.735

The structures presented in Figure 1 indicate that the iron atom is located above the planar structure of graphene, in agreement with the other extensive models reported in the literature. The elevation of the iron atom of the substrates is shown in Table S3. In the case of Fe-0N and Fe-3N, the elevation reported in this work was 1.40 and 1.28 Å, respectively. Gao and Yang obtained an elevation of 1.35 and 1.23 Å for Fe-0N and Fe-3N.^35−36,37,38^ Krasheninnikov et al. presented an elevation close to 1.40 Å^39^ for Fe-0N. According to our calculations, the distance between carbon atoms in the pristine graphene is 1.41 Å as expected.^40^ The presence of the iron atom distorts the honeycomb structure of graphene and forms bonds with the nearest neighboring carbon atoms, with a length of 1.78 Å. This distance slightly elongates from 1.78 to 1.80 Å, and the elevation of the iron atom decreases in configurations with pyridinic N–C bonds (see Table S3) as mentioned above. The distortion of the graphene structure due to the presence of an iron atom is also reported in the literature. Chen obtained a bond length of 1.76 and 1.77 Å for the Fe–C and Fe–N bonds, respectively.^20^ Yang reported very similar results, 1.762 A and 1.78 Å for the Fe–C and Fe–N bonds, respectively.^14^ A bond length of 1.76 Å is presented for the Fe–C bond by Gao et al.^38^ The Bader charge analysis reveals that the iron atom loses charge in relation to the number of nitrogen atoms from 0.73 *e* (Fe-0N) to 0.98 *e* (Fe-3N) which is related with the difference in electronegativity of the atoms (see Table S3). It is higher for nitrogen than for iron and carbon. This charge loss has been reported in the literature, such as the work of Chen et al., where a charge variation of 1.06 *e* for Fe-0N to 1.14 *e* for Fe-3N is obtained.^20^ Gao et al. calculated 0.69 *e* (Fe-0N) to 0.89 *e* (Fe-3N).^35,38^

Interestingly, when pyridinic-N sites are increased, the charge transference between the iron atom and nitrogen atoms increases. This, in principle, is indicative of a stronger interaction. However, the binding energy becomes more positive. This apparently is contradictory, since a greater interaction between these atoms should give greater stability to the substrate. This effect has been reported in various works^20,36,41^ as mentioned above without a convincing explanation. Kropp et al.^42^ analyzed the binding energy of graphene codoped with transition metals and nitrogen. In the case of a single vacancy, the binding energy of most graphene sheets doped only with a single transition metal atom is reported to be negative (exothermic binding process). On the other hand, this binding energy increases (less exothermic) by modifying the coordination of the metal atom with a nitrogen atom. This is explained by the fact that the energy required to form a monovacancy is lower in the presence of a pyridinic-N site. Kropp et al. reported that the single vacancy formation energy is 753 kJ/mol for pristine graphene. But this energy was 474 kJ/mol when a pyridinic-N site is present. This has an effect on the binding energy of the iron atom which is −235 kJ/mol single vacancy and close to −100 kJ/mol single vacancy with a pyridinic-N site. This means that the substitution of a carbon atom for a nitrogen atom makes the bonding of the iron atom less favorable energetically, even while remaining exothermic. In that same sense, Li et al. showed that the energy required to dope a single vacancy graphene sheet with one (−4.23 eV), two (−3.49 eV), and three (−2.59 eV) nitrogen atoms increases.^43^ The negative value indicated the doping was thermodynamically favorable, but when more nitrogen atoms were present, the process required more energy. Therefore, although a strong interaction with iron is observed by the nitrogen atoms, these N atoms destabilize the substrate as a whole, which is the reason why the binding energy increases.

The electronic structure was analyzed by using PDOS. Figure S1a shows narrow overlapping peaks corresponding to the Fe 3d and C 2p orbitals near the Fermi energy. This indicates a strong hybridization between the iron and its neighboring atoms. When the coordination with nitrogen atoms is modified, the hybridization remains strong; however, the interaction extends to the surrounding carbon atoms (see Figure S1b–d). Figure 1 shows the CDD plots (isosurface ≈0.003 e/bohr^3^) revealing a concentration of charge in the region close to the iron atom and its neighborhood. Some remaining charge is also detected on each carbon with a p_*z*_-lobe shape. The shape of the charge distribution for the surrounding carbon atoms changes when the coordination is modified. The results shown are quite similar to those reported by Chen et al.^20^ even though they used a code based on the utilization of plane-wave basis set.

It is also important to understand the interaction of the 3d electrons of the iron atom with the rest of the substrate. For this purpose, the GGA+*U* method was used.^32^ The effect of increasing the *U*–*J* value on the electronic structure of Fe-0N was verified, and it was noted that for values greater than 1 eV, the Fe-0N substrate presents a magnetic moment different from zero. According to what was found in the literature, the magnetic moment of the Fe-0N substrate must be zero.^39,44^ That is why, *U*–*J* = 1 eV was used as the appropriate value for*U*–*J*. Figure S2 provides the density of states of Fe-0N and Fe-3N. In this case, the differences are quite minimal in relation to the results obtained without using GGA+*U*, which is also reported in the literature.^45,46^

### CO_2_ Adsorption on the Substrates

The adsorption of the CO_2_ molecule was thoroughly examined. Initially, the molecule was arranged in a side-on (s) configuration to the graphene plane (xy plane) in six different orientations; each of them rotated 30° with respect to the other, covering the points of symmetry of the triangle formed by the neighbor iron atoms (see Figure S3). Then, the CO_2_ molecule was also placed in an end-on (e) configuration to the plane of the graphene (perpendicular to the graphene plane). In order to have a greater range of analysis, the molecule was deflected by a small angle in relation to the *z* axis and toward the points of symmetry of the parallel configuration. There were a total of 13 initial configurations for the CO_2_ molecule for each coordination type. In Figure S3, the six initial configurations for the case of Fe-0N in the side-on orientation. The structures were relaxed for all of these initial configurations.

They were compared to each other in terms of energy, and the most stable ones were chosen to characterize the adsorption. This type of analysis has not been described at this level of detail in the literature, to the best of our knowledge.

### Geometric Property, Bader Charge Analysis, and Stability of CO_2_ Adsorption

**Figure 2:**
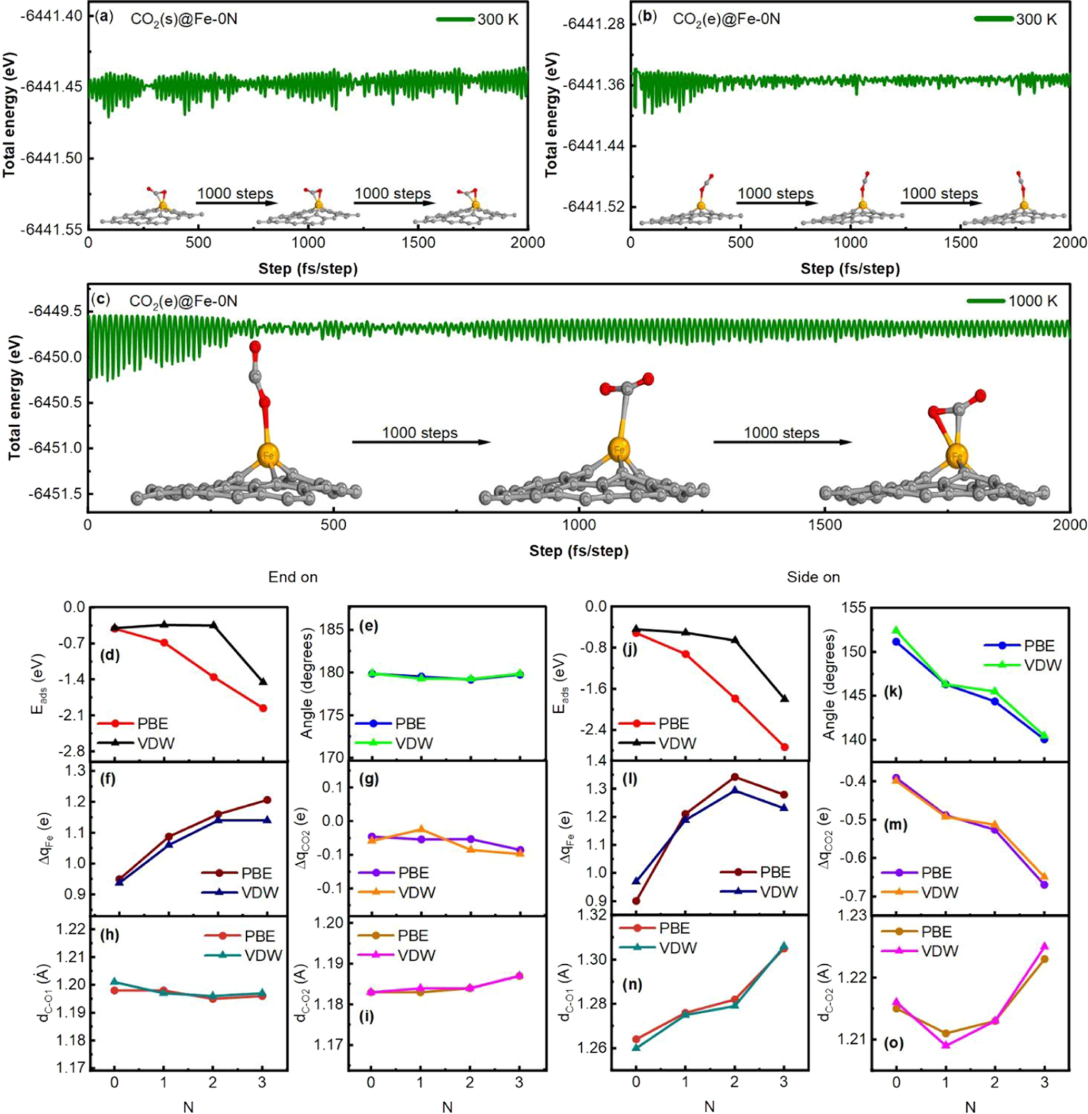
Molecular dynamics for CO_2_(*s*)@Fe-0N (a) and CO_2_(*e*)@Fe-0N (b) at 300 K and (c) CO_2_(*e*)@Fe-0N at 1000 K. Adsorption energy (*E*_ads_), charge transfer, and geometric parameter curves versus added nitrogen atoms, in end-on and side-on configuration. Adsorption energy (*E*_ads_) (d, j), CO_2_ angle (e, k), Δq_Fe_ (f, l), Δq_CO2_ (g, m), C–O1 bond length (h, n), and for C–O2 length (i, o) in angstrom.

The real-life application of the system is dependent on its structural stability. One way to verify this is through thermal stability analysis. Therefore, ab initio molecular dynamics (AMD) calculations have been used. The Nosé thermostat^47^ was used to control the temperature and a time step of 0.5 fs as the numerical integration step of the equations of motion. Figure 2a–c shows the variation of the total energy in relation to the time of CO_2_(*s*)@Fe-0N at 300 K, CO_2_(*e*)@Fe-0N at 300 K, and CO_2_(*e*)@Fe-0N to 1000 K, respectively. It can be seen in the three figures that the energy fluctuates around a point. Furthermore, the evolution of the structure during the trajectory was examined. It was found that the substrate remained stable and the adsorption of the molecule was not interrupted at room temperature (300 K). Figure S4 shows that the structure of the CO_2_(*e*)@Fe-0N system remains stable at 500 K. When analyzing the trajectory of CO_2_(*e*)@Fe-0N at 1000 K, a transition from the end-on to side-on configuration is observed. There is an animation of this transition in Supporting Information and it is available online. This indicates that the structures of the systems studied remain stable and that adsorption is sustained even at high temperatures. It should be noted that the thermal stability of the substrates has been examined in the literature and is preserved at high temperatures (around 700 K).^20,48^

Figure 2d–o depicts the changes in the adsorption energy, charge transfer, and geometric characteristics with the pyridinic-N coordination. The CO_2_ adsorption simulation in the end-on and side-on configurations was carried out with the PBE and vdW functionals. According to the curves, the adsorption energy decreases with the change of coordination from −0.41 to −1.96 eV for CO_2_(*e*)@Fe-3N in the end-on adsorption with PBE (Figure 2d). It is also evident that *E*_ads_ decreases with coordination, indicating better adsorption. The highest and lowest adsorption energies obtained for CO_2_(*s*)@Fe-0N and CO_2_(*s*)@Fe-3N are −0.52 and −2.73 eV, respectively (see Figure 2j). The *E*_ads_ in decreasing behavior suggests that side-on configuration is more favorable than end-on in the adsorption.

The adsorption energy (*E*_ads_) is also modified due to the pyridinic-N doping with the vdW method. *E*_ads_ decreases from −0.40 eV for 0N to −1.46 eV for 3N for the end-on configuration. It can be noted in Figure 2d that the *E*_ads_ values are nearly equal for the CO_2_(*e*)@Fe-1N and CO_2_(*e*)@Fe-2N configuration. It can be seen in Figure 2j that the adsorption energy curve of the side-on configuration follows the same tendency as it does for PBE, also suggesting that side-on geometry is more favorable for CO_2_ adsorption.

Figure 2 shows the effect of the change in coordination on the structure of the molecule. It is noted that the CO_2_ structure is altered, especially in the side-on configuration with slight changes in the end-on configuration. The estimated C–O bond length is 1.19 Å for the isolated molecule, but after adsorption, the structure turns slightly asymmetric (Figure 2h,i,e). The C–O2 bond length decreases by about 0.6% while the C–O1 bond length slightly increases (∼0.7%). The end-on adsorption structure has geometric characteristics similar to those found by Promthong et al.^18^ for nanoflake geometric model similar to the CO_2_@Fe-0N configuration. In his work, Promthong obtained a bond length of 1.97 and 1.184 Å for C–O1 and C–O2, respectively. It was determined to be 1.198 and 1.184 Å for C–O1 and C–O2, respectively, according to our results. Lv et al. reported a similar configuration for the adsorption of CO_2_ on a MgO surface, the C–O1 bond (1.174–1.177 Å) is slightly larger than the C–O2 bond (1.173–1.176 Å) and a linear shape. The charge transfer for the CO_2_ molecule, obtained by using the Bader charge analysis for end-on configuration, with the PBE method is shown in Figure 2g. Figure 2g indicates values between −0.03 *e* and −0.06 *e*, which is characteristic of physisorption according to Lv et al., who found values from −0.01 to −0.05*e.*^49^ In the VDW method for end-on configuration, there is a slight asymmetry in the C–O1 and C–O2 bond lengths, as in PBE. C–O1 and C–O2 increases and decreases, respectively, as is shown in Figure 2h,i,e. The geometry remains linear, and no bending or distortion is observed since its angle is nearly around 180° (see Figure 2e). The change transfer to the CO_2_ molecule gives values between −0.041 and −0.068 eV, suggesting that it is a physisorption process.

A slight elongation is noted in the C–O1 bond (from 1.26 to 1.30 Å) and the O1–C–O2 angle becomes more acute (from 151.15 to 140.06°) for the CO_2_(*s*)@Fe-0N and CO_2_(*s*)@Fe-3N configuration, respectively (see Figure 2k,n,o). The C–O2 bond length also increases from 1.21 to 1.22 Å but not monotonically (see Figure 2o). It should be noted that the elongation of the length of the CO_2_ bonds as well as the decrease of the bending angle is a well-known characteristic of CO_2_ activation^50^. This flexed form of the molecule has been reported in several works that study the chemisorption of CO_2_. For example, Ding et al.^51^ obtained angles from 162° to 126° and an increase in the C–O1 and C–O2 bonds from 1.192 to 1.28 Å for the deformation of the molecule when a chemisorption occurs. Lv et al.^49^ found 123.94°, 1.387, and 1.213 Å for the angle, C–O1, and C–O2, respectively, in the case of chemisorption. The charge acquired by the CO_2_ molecule during adsorption in the side-on configuration is indicated in Figure 2m. Bader’s population analysis indicates that the charge that CO_2_ gains increases with the modification of coordination, reaching 0.67 *e* for the CO_2_(*s*)@Fe-3N configuration. The modification of the angle of the O1–C–O2 bond is related to the charge gain and the activation of the molecule. The bending angle is 135° when CO_2_ gains 1 *e* of charge.^50^ Thus, the charge gained by CO_2_ in side-on adsorption is much higher than that in the end-on case. The structure of the CO_2_(*s*)@Fe-3N configuration was analyzed with the vdW method, see Figure 2n,k,o. The C–O1 bond modifies its length from 1.26 to 1.24 Å. The geometry of the molecule is no longer linear, it suffers a bending due to change in N coordination and becomes more acute (see Figure 2k); this might be due to CO_2_ molecule activation. The structural changes in the structure are quite similar to those obtained with PBE from Fe-0N to Fe-2N. However, when the adsorption occurs on Fe-3N, it can be seen that the C–O1 bond is shortened instead of elongated, and the C–O2 bond is not elongated as in the case of PBE (see Figure 2n,o). This difference will be better explained in section Evolution of Adsorption Characteristics Change of Pyridinic-N Coordination. The charge gained by the molecule goes from −0.399 to −0.649 e. This charge gain is also reported in the work of Din et al. and depending on the adsorption configuration on the Ni surface this charge gain was from 0.93e to 0.62e.^51^ For Lv et al., the molecule obtains a charge of 0.88e. Note that gained charge in side-on configuration is higher than in the end-on configuration, so the former is the most stable structure as with PBE.

**Figure 3:**
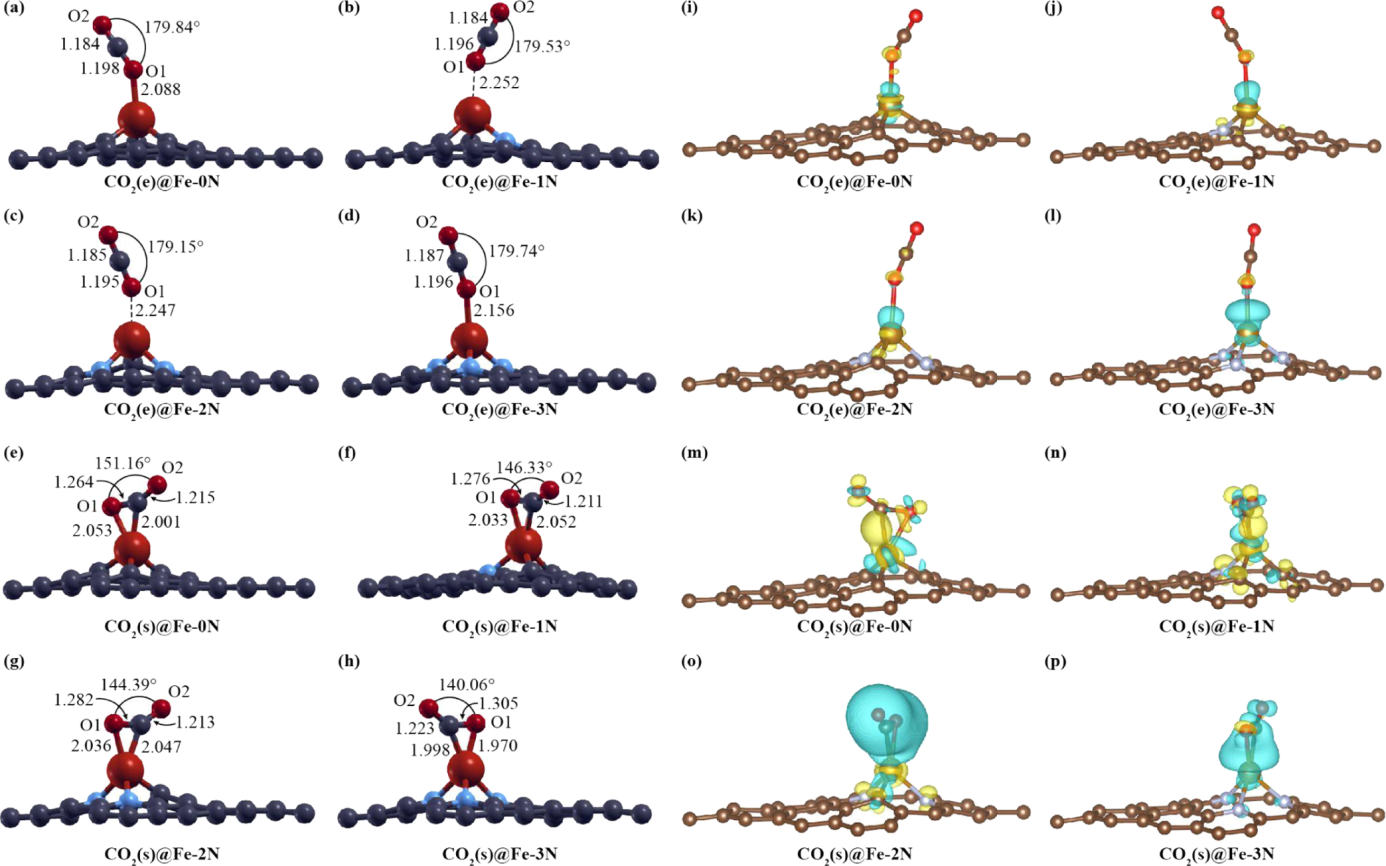
Relaxed structures for CO_2_(*e*)@Fe-0N (a), CO_2_(*e*)@Fe-1N (b), CO_2_(*e*)@Fe-2N (c), CO_2_(*e*)@Fe-3N (d), CO_2_(*s*)@Fe-0N (e), CO_2_(*s*)@Fe-1N (f), C*O*_2_(*s*)@Fe-2N (g), and CO_2_(*s*)@Fe-3N (h) configurations. Charge density difference for CO_2_(*e*)@Fe-0N (i), CO_2_(*e*)@Fe-1N (j), CO_2_(*e*)@Fe-2N (k), CO_2_(*e*)@Fe-3N (l), CO_2_(*s*)@Fe-0N (m), CO_2_(*s*)@Fe-1N (n), CO_2_(*s*)@Fe-2N (o), and CO_2_(*s*)@Fe-3N (p) (isosurface = 0.003 e/bohr^3^) configurations.

Figure 3i–l shows the CDD plots (isosurface = 0.003 e/bohr^3^) for the end-on adsorption mode in which a low charge distribution between CO_2_ and the substrate is revealed to slightly increase with coordination. The CO_2_(*e*)@Fe-3N presents the highest charge density between the adsorbent and the adsorbate among the other systems (see Figure 3l). Figure 3m–p shows the CDD plots of side-on adsorption of the CO_2_ molecule. It is clearly noted that redistribution of charge occurs between the molecule and the iron atom, and it changes with coordination. The CO_2_(*s*)@Fe-3N configuration shows the most distributed charge density (see Figure 3p). The CDD plots indicate a more covalent interaction, as pyridinic-N coordination increases for side-on adsorption.

An interesting issue to discuss is the capture process of the CO_2_ molecule. This is not discussed as much in the literature as we have been able to learn. First, as we have seen from Table S2, the charge of the iron atom decreases with the increase in the number of pyridinic-N sites in the substrate. This is expected since nitrogen is more electronegative than carbon, as discussed in the previous section. However, the molecule gains charge after it is captured and activated by the substrate. Then, activation is stronger when there are more pyridinic-N sites. This apparently is contradictory because the origin of the charge that activates the molecule should come from an electron-deficient atom. To clarify this, we have two important factors to take into account. One is the oxidation state of iron, and the other is the electronegativity of oxygen. As is known, the most common oxidation states of iron are +2 and +3, so in this case, as in many others, iron’s tendency is to donate electrons, especially when it interacts with more electronegative elements. On the other hand, oxygen is the most electronegative element in the entire system. That is why the molecule has the ability to accumulate charge with an increase of valence electrons by the nitrogen atoms. This implies that there is a combined effect between both factors so that activation of the molecule is achieved. In fact, a similar result is obtained by negatively charging the substrate, as reported by Li et al., when the charge density of a divacancy graphene sheet doped with one iron atom and four pyridinic-N sites increases. The charge transferred to the CO_2_ molecule increases with charge density.^52^ The same effect is observed in the work of Tabandeh^53^ and He^54^ who study the capture of CO_2_ in graphdiyne and graphyne, respectively, decorated with transition metals. It is reported that adsorption is related to a charge transfer from the substrate to the molecule (which is negatively charged) and a loss of charge on the part of the metal (positively charged).

In molecular dynamics of CO_2_(*e*)@Fe-0N, at 1000 K, a transition to CO_2_(*s*)@Fe-0N is observed that corresponds to an energy equal to 0.086 eV that should correspond to a barrier energy. Jiao et al. found that the transition from physisorption to chemisorption states of carbon dioxide has a barrier height of 0.82 eV on a hexagonal graphitic boron nitride single layer.^55^ He et al. build a single-layer graphyne embedded with different single atoms, the energy barrier of physisorption to chemisorption of CO_2_ varies between 0.02 and 0.29 eV.^54^ It is necessary to study more to obtain many stable states in order to study the transition from end-on to side-on configuration.

### Electronic Structure and Chemical Analysis of the CO_2_ Adsorption on Single Vacancy Iron-Doped Graphene

**Figure 4:**
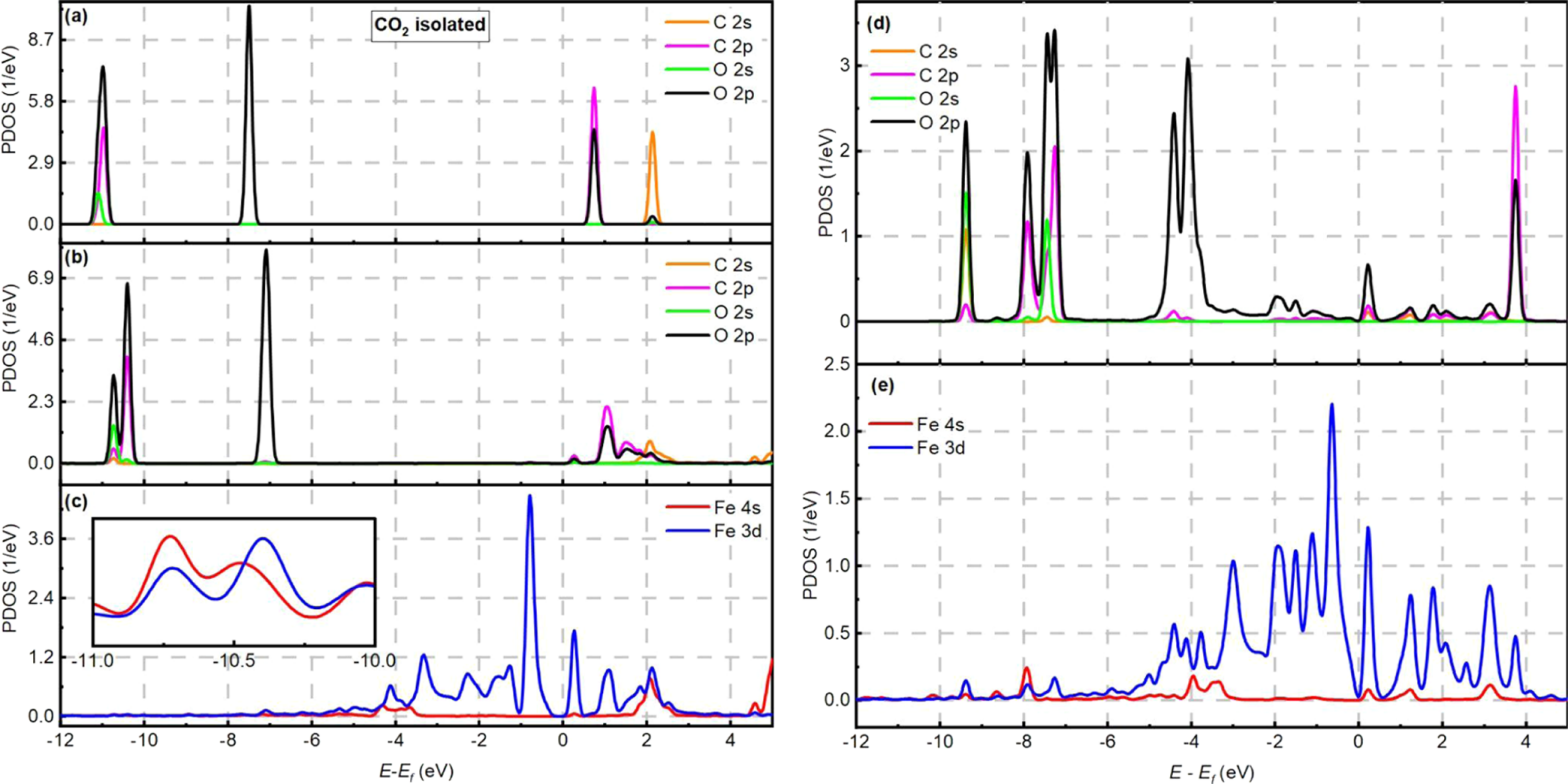
PDOS curves of isolated CO_2_ (a), CO_2_ (b), and Fe atom (c) in CO_2_(*e*)@Fe-0N configuration. PDOS curves of CO_2_ (d) and Fe atom (e) in the CO_2_(*s*)@Fe-0N configuration.

Figure 4a shows a PDOS analysis of the valence orbitals of the CO_2_ molecule before adsorption. For the CO_2_(*e*)@Fe-0N configuration (see Figure 4b), four well-defined peaks can be seen in the energy range (−12.0 eV, 5 eV), two in the occupied, and two in the unoccupied states (see also Figure S5 for a series of additional peaks to compare). After end-on adsorption in the CO_2_(*e*)@Fe-0N system (see Figure 4b), the peak at −11 eV (isolated CO_2_) splits into two close peaks and shifts to the positive direction. Furthermore, after the Fermi energy, more additional peaks appear, indicating some hybridization of the CO_2_ molecule with the substrate states. Remarkably, the PDOS of the Fe 4s and Fe 3d orbitals shows little hybridization with the CO_2_ orbitals below the Fermi energy. In fact, as shown in Figure 4c (inset), the iron peaks are quite small even though there is resonance with the CO_2_ peaks. The resonant peaks are at −10.6 eV (O 2s 2p, Fe 4s 3d), −10.3 eV (O 2s, C 2p, Fe 3d), −7.5 eV (O 2p, Fe 4s 3d), 0.25 eV (O 2s 2p, C 2p, Fe 4s 3d), 1.2 eV (O 2p, C 2p, Fe 3d), and 2.1 eV (O 2p, C 2s 2p, Fe 4s 3d).

The PDOS curves for the CO_2_(*s*)@Fe-0N system are also shown in Figure 4d. Eight peaks can be seen in the PDOS curve of the CO_2_ orbitals after side-on adsorption, together with smaller peaks near the Fermi energy. The number of peaks is evidently higher compared to the peaks found in the CO_2_(*s*)@Fe-0N configuration, suggesting a greater interaction with the substrate. Figure 4e presents the PDOS curves of the Fe 4s and Fe 3d orbitals. In Figure 4d,e the resonance with the CO_2_ orbitals is clearly observed. These resonant peaks are found at −9.4 eV (O 2s 2p, C 2s 2p, Fe 3d 4s), −7.9 eV (O 2s 2p, C 2s 2p, Fe 3d 4s), −7.45 eV (O 2s 2p, C 2p, Fe 3d), −7.25 eV (O 2p, C 2p, Fe 3d), −4.4 eV (O 2p, Fe 3d 4s), −4.1 eV (O 2p, Fe 3d), 0.25 eV (O 2s 2p, C 2s 2p, Fe 3d 4s), and 3.75 eV (O 2p, C 2p, Fe 3d).

The PDOS curves of the CO_2_(*e*)@Fe-0N and CO_2_(*s*)@Fe-0N systems obtained with the vdW functional were also analyzed. In Figure S6a,c, it can be seen that the changes are minimal compared to the results obtained with PBE. In the case of the CO_2_(*e*)@Fe-0N system, we can notice the three occupied states of CO_2_ that hybridize with the iron states, as seen in Figure 4b. A slight difference is perceived in states near and above the Fermi energy. In the case of the CO_2_(*s*)@Fe-0N configuration, the differences are almost not noticeable (see Figure S6b,d). The peaks are in the same positions, and the same number of peaks is maintained. It can be inferred that the nonlocal correction of the vdW functional does not have a determining effect when the adsorption occurs on the Fe-0N substrate.

**Figure 5:**
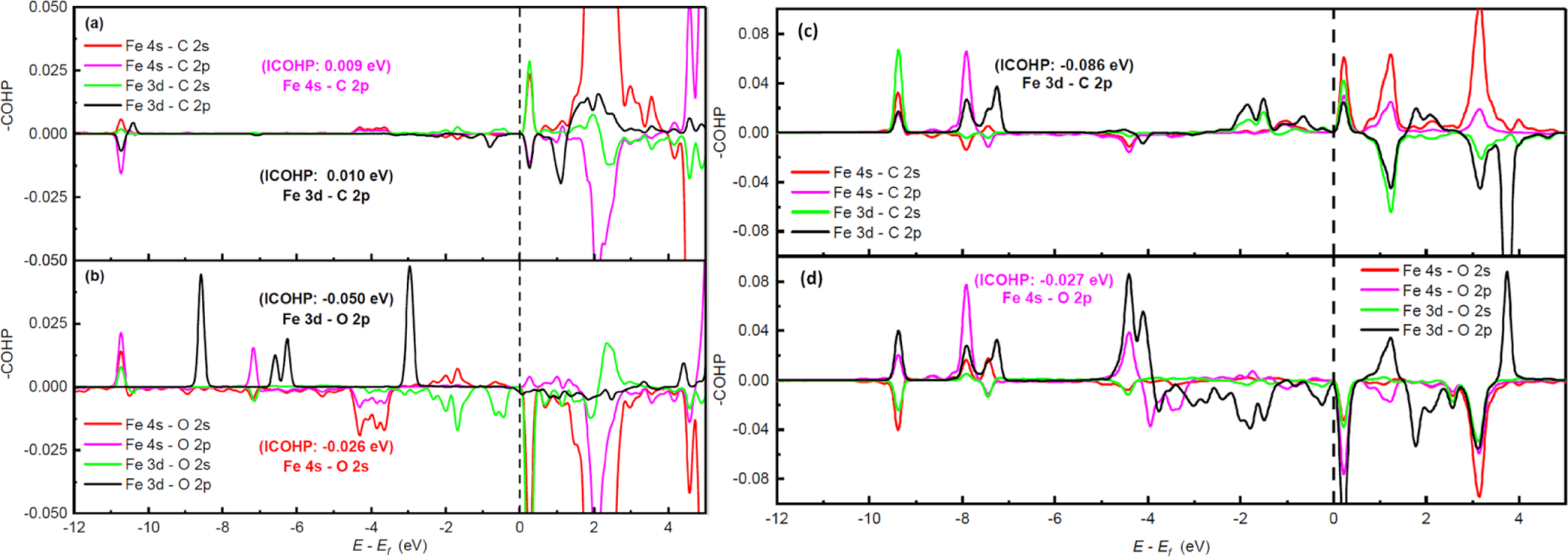
Crystal orbital Hamilton population (COHP) curves of Fe–C (CO_2_) (a) and Fe–O (b) orbital pairs in the C*O*_2_(*e*)@Fe-0N configuration. The total ICOHP values for Fe–C and Fe–O are 0.079 and −0.147 eV, respectively. COHP curves of Fe–C (CO_2_) (c) and Fe–O (d) orbital pairs in C*O*_2_(*s*)@Fe-0N configuration. The total ICOHP values for Fe–C and Fe–O are −0.249 and −0.076 eV, respectively.

The −COHP curves are presented in Figure 5a,b and analyzed to understand the interaction of CO_2_ and the substrate from a chemical point of view in the CO_2_(*e*)@Fe-0N configuration. Note that the bonding and antibonding states coincide with the PDOS peaks presented in Figure 4b,c. The orbital pairs Fe 4s-C 2p and Fe 3d-C 2p located at −10.6 eV are the ones that most contribute to the Fe–C antibonding interaction (Figure 5a). On the other hand, there is an important number of bonding states corresponding to the Fe–O interaction in Figure 5b. The contribution of the Fe 3d-O 2p orbital pair is highlighted, which is the only one that does not have antibonding states below the Fermi energy. Moreover, note a region of antibonding states between −4.5 and −3 eV corresponding to the Fe 4s-O 2s orbital pair contributing less than the Fe 3d-O 2p orbital pair. This is reflected in the ICOHP values. In Figure 5a, the peaks corresponding to the interaction between iron and carbon (CO_2_) are quite small (ICOHP = 0.079 eV), meaning that the interaction is low. Nevertheless, the positive value of the ICOHP indicates, albeit a slight, antibonding interaction. For the Fe–C interaction, the greatest contribution is given by Fe 4s-C 2p (ICOHP = 0.009 eV) and Fe 3d-C 2p (ICOHP = 0.01 eV). Figure 5b presents the chemical interaction between oxygen and iron (ICOHP = −0.147 eV). The resonant peaks below the Fermi energy in the PDOS correspond to the interaction between these two atoms, while Fe 3d-O 2p (ICOHP = −0.050 eV) and Fe 4s-O 2s (ICOHP = −0.026 eV) are the major contributors to the bonding interaction. As discussed, the adsorption in the C*O*_2_(*e*)@Fe-0N configuration does not cause significant changes in the geometry of the molecule but causes a few modifications in the electronic structure. In addition, since the adsorption energy is low, the interaction between CO_2_ and the substrate in the C*O*_2_(*e*)@Fe-0N configuration is considered physisorption.

Figure 5c,d shows the −COHP curves of the contributions of the Fe–C and Fe–O bonds in the C*O*_2_(*s*)@Fe-0N configuration, in which a match is noted in the positions of the bonding and antibonding peaks with the peaks shown in Figure 4d,e. Fe and C are clearly bonded since most of the contributions of the orbital pairs below the Fermi energy are in the bonding states (Figure 5c). Antibonding hybridization between Fe and O is observed near the Fermi energy (between −4 and 3.5 eV) in Figure 5d. This causes the Fe–C interaction (ICOHP = −0.249 eV) to be stronger than the Fe–O interaction (ICOHP = −0.076 eV) in the C*O*_2_(*e*)@Fe-0N configuration. Thus, side-on adsorption favors interaction with the carbon atom. In addition, the Fe–O bonding, although weaker than the Fe–C bond, stabilizes the adsorption by having bonding character. It is interesting to note the contribution of antibonding states between −4.0 eV up to the Fermi energy which are associated mainly with the Fe 3d-O 2p orbital pair. This was not observed in the C*O*_2_(*e*)@Fe-0N configuration, in which the Fe 3d-O 2p orbital pair is the most important. On the other hand, three peaks are associated with the bonding states corresponding to the Fe 4s-O 2p orbital pair at −9.4, −7.9, and −4.4 eV. This makes the contribution of this orbital pair larger than that of the Fe 3d-O 2p orbital pair, implying that the side-on arrangement favors the sigma character of the Fe–O1 bond in the C*O*_2_(*s*)@Fe-0N configuration.

The orbital pairs that most contribute to the Fe–C and Fe–O bonds are Fe 3d-C 2p (ICOHP = −0.086 eV) and Fe 4s-O 2p (ICOHP = −0.027 eV), whereas the Fe 3d-O 2s orbital pair (ICOHP = 0.007 eV) has antibonding characters for the case of the Fe–O bonds, as in the end-on adsorption case. Moreover, Fe 3d-C 2s (ICOHP = −0.043 eV), Fe 4s-C 2p (ICOHP = −0.019 eV), and Fe 4s-C 2s (ICOHP = −0.019 eV) also contribute to the stability of the Fe–C bond in contrast to the end-on adsorption in which all of these orbitals are antibonding. As mentioned above, the contribution of the Fe 3d-O 2p orbital pair to the Fe–O1 bond is low (ICOHP = −0.008 eV) in the C*O*_2_(*s*)@Fe-0N configuration. Note that the contribution of the Fe 4s-O 2s orbital pair (ICOHP = −0.008 eV) is also low. The adsorption in the C*O*_2_(*s*)@Fe-0N configuration has chemisorption characteristics and also has effects on the appearance of new peaks in the PDOS of the molecule and the bending of the geometry. However, the charge transferred to CO_2_ (−0.39 *e*) is not enough to complete the chemisorption, which is also reflected in the low adsorption energy. Because of this, the substrate does not fully chemisorb the molecule. However, it should be noted that the interaction is much greater than in the case of the *CO_2_(e*)@Fe-0N configuration.

Chemical analysis was also performed to study the adsorption using vdW. The COHP curves for the C*O*_2_(*e*)@Fe-0N and C*O*_2_(*s*)@Fe-0N configurations are presented in Figure S7. In this case of the C*O*_2_(*e*)@Fe-0N system, it is observed that the most significant change is near the Fermi energy. Figure S7a,b shows a shift below the Fermi energy of the states that PBE established as unoccupied (see Figure 5a,b). Otherwise, the behavior of the other states is maintained up to −12 eV. By calculating the ICOHP values, a decrease in the interaction of the orbital pairs Fe 3d-C 2p (ICOHP = 0.005 eV), Fe 4s-C 2p (ICOHP = 0.013 eV), Fe 3d-O 2p (ICOHP = −0.017 eV), and Fe 4s-C 2s (ICOHP = −0.010 eV) is recognized. On the other hand, as expected, the greatest interaction occurs with the oxygen atom (O1). In the case of the C*O*_2_(*s*)@Fe-0N system, the greatest changes occur in the Fe 3d-C 2p orbital pair (ICOHP = 0.01 eV), which presents a series of antibonding states from the Fermi energy to −3.0 eV (see Figure S7c). The other orbital pairs present minor differences. For example, the Fe 4s-O 2s orbital pair (ICOHP = −0.026 eV) barely differs from that calculated by PBE (Figure 5d).

### Electronic Structure and Chemical Analysis of the CO_2_ Adsorption on Pyridinic-N Iron-Doped Graphene

**Figure 6:**
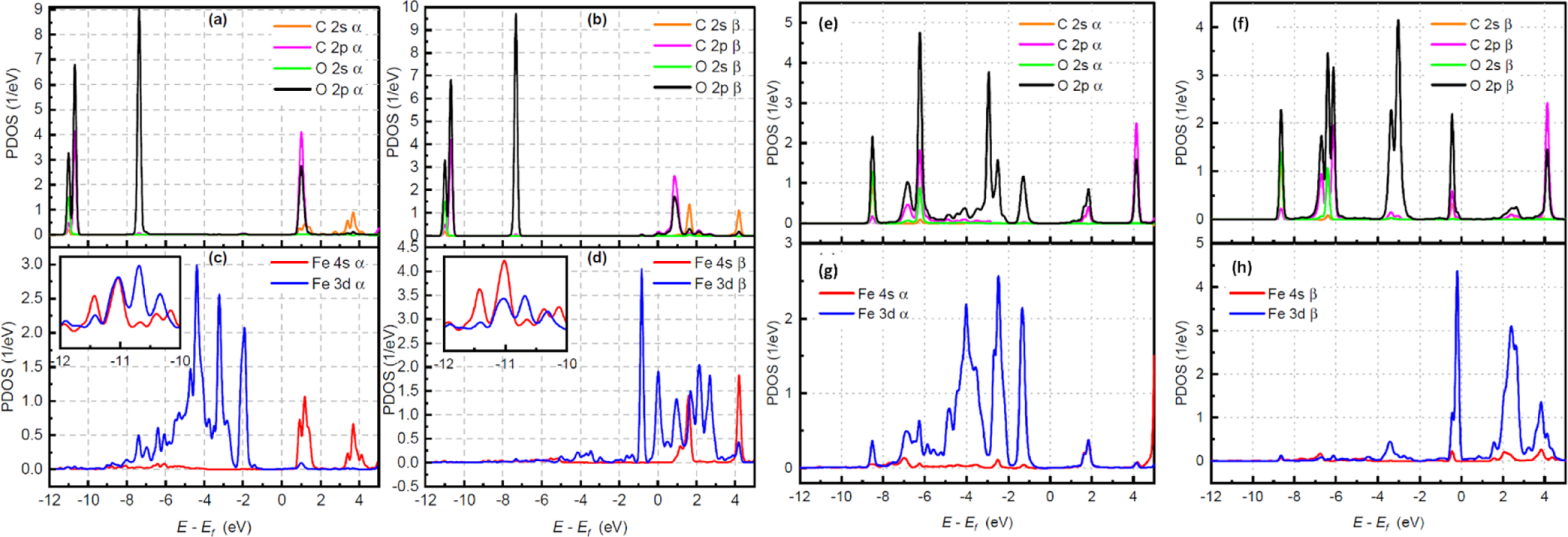
PDOS curves of CO_2_ majority (a), minority (b), Fe atom majority (c), and minority (d) for CO_2_(*e*)@Fe-3N configuration. The inset plots show the small Fe 3d and 4s peaks in resonance with the CO_2_ peaks. PDOS curves of CO_2_ majority (e), minority (f), Fe atom majority (g), and minority (h) for CO_2_(*s*)@Fe-3N configuration.

Modification of the pyridinic N coordination results in changes in the electronic structure of the adsorbed system. To compare the changes with the alteration in coordination, the electronic structure of the CO_2_(*e*)@Fe-3N system was examined through PDOS analysis. The three main peaks corresponding to the occupied states remain almost unchanged in relation to the CO_2_(*e*)@Fe-0N system. The most noticeable changes are in the states above the Fermi energy, which due to the interaction with the iron states show spin polarization (see Figure 6a,b). The Fe 4s and Fe 3d states cause high spin polarization of the adsorbed system, as observed in Figure 6c,d. In contrast, they hybridize poorly with the CO_2_ states, as in the case of the CO_2_(*e*)@Fe-0N system. The inset plots in Figure 6c,d show the small peaks of the hybridization for the iron states with the molecule states. The peaks that correspond to occupied states have barely changed with the coordination change. The occupied states shift negatively by almost 0.5 eV in contrast to the unoccupied states (mostly C 2s states), which present splits.

Figure 6e,f shows eight peaks in the PDOS of CO_2_ with the majority and minority states in the CO_2_(*s*)@Fe-3N configuration. The resonance between the CO_2_, Fe 4s, and Fe 3d states is noted when looking at Figure 6g,h. The resonant peaks for the majority states are −8.52 eV (O 2s 2p, C 2s 2p, Fe 3d), −6.84 eV (O 2s 2p, C 2p, Fe 3d 4s), −6.24 eV (O 2s 2p, C 2s 2p, Fe 3d 4s), −4.10 eV (O 2p, Fe 3d), −2.51 eV (O 2p, Fe 3d 4s), −1.30 eV (O 2p, C 2p, Fe 3d 4s), 1.84 eV (O 2p, C 2s 2p, Fe 3d 4s), and 4.14 eV (O 2p, C 2p, Fe 3d 4s). The positions of the minority state peaks are −8.75 eV (O 2s 2p, C 2s 2p, Fe 3d 4s), −6.71 eV (O 2p, C 2p, Fe 3d 4s), −6.13 eV (O 2p, C 2p, Fe 3d), −3.36 eV (O 2p, C 2p, Fe 3d), −3.02 eV (O 2p, Fe 3d), −0.44 eV (O 2p, C 2s 2p, Fe 3d 4s), 2.53 eV (O 2p, C 2s 2p, Fe 3d), and 4.13 eV (O 2p, C 2p, Fe 3d). Compared to CO_2_(*s*)@Fe-0N, there is no increase in the number of peaks but a strong correlation with the spin polarization of the iron atom. For example, there are three peaks between −8.0 and −7.0 eV in the configuration CO_2_(*s*)@Fe-0N (see Figure 4d). In Figure 6e, only two peaks of majority states can be seen between −7.4 and −5.7 eV; however, the three peaks in Figure 6f remain for the minority states between −7.0 and −5.8 eV. There is also a positive shift (from 2 to 1 eV) for all of the CO_2_ peaks in the CO_2_(*s*)@Fe-3N configuration in relation to the CO_2_(*s*)@Fe-0N one. Eventually, there is an additional majority (minority) state peak at −1.30 eV (−0.44 eV) in Figure 6e (Figure 6f), which comes from splitting of the peak located at −4.1 eV in Figure 4d.

As in the case of adsorption on the Fe-0N substrate, we also analyzed the changes in the PDOS curves of adsorption on Fe-3N using the vdW functional (see Figure S8). When observing the PDOS curves, no significant changes are seen in either the end-on or side-on orientations. The same number of peaks and almost the same positions are maintained. Some peaks are shifted, such as the majority (α) state, which is located at −2 eV (in Figure 6c) and corresponds to the CO_2_(*e*)@Fe-3N configuration. That state shifts toward the Fermi energy by less than 0.5 eV (Figure S8c). However, most states remain unchanged.

**Figure 7:**
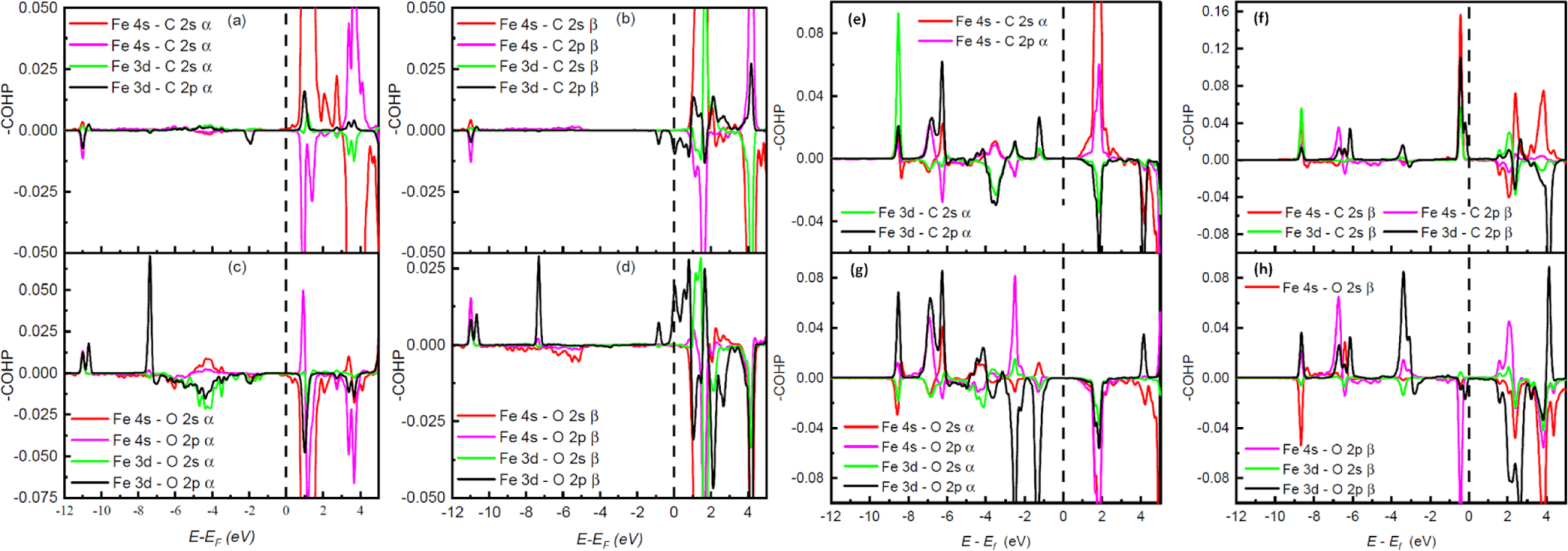
Crystal orbital Hamilton population (COHP) curves of majority Fe–C (CO_2_) (a), minority Fe–C (CO_2_) (b), majority Fe–O (c), and minority Fe–O (d) orbital pairs in CO_2_(*e*)@Fe-3N configuration. The total ICOHP values for Fe–C and Fe–O are 0.079 and −0.102 eV, respectively. COHP curves of majority Fe–C (CO_2_) (e), minority Fe–C (CO_2_) (f), majority Fe–O (g), and minority Fe–O (h) in CO_2_(*s*)@Fe-3N configuration. The total ICOHP values for Fe–C and Fe–O are −0.245 and −0.110 eV, respectively.

Figure 7a–d shows the −COHP plots of the contributing orbital pairs of the Fe–C and Fe–O bonds for the CO_2_(*e*)@Fe-3N configuration. In Figure 7a,b, the main peaks at −11 eV correspond to antibonding interactions between the Fe and C orbitals. The Fe 4s-C 2p and Fe 3d-C 2p orbital pairs are the major contributors to the antibonding majority and minority states located at −11 eV, which is similar to the case for the CO_2_(*e*)@Fe-0N configuration. There is also an antibonding majority (minority) state corresponding to the Fe 3d-C 2p orbital pair at −1.92 eV (−0.85 eV). These two peaks result from the splitting of the −0.8 eV peak in Figure 5a due to spin polarization of the Fe 3d orbitals. This state causes the Fe 3d-C 2p orbital pair to outperform Fe 4s-C 2p in contributing to the Fe–C interaction in the same way as in the case of the CO_2_(*s*)@Fe-0N configuration.

In the case of the Fe–O interaction, a series of antibonding majority states between −7 and −1.5 eV are noted, which correspond to the Fe 3d-O 2p orbital pair (see Figure 7c). This reduces the total contribution of the Fe 3d-O 2p orbital pair compared to the CO_2_(*e*)@Fe-0N configuration where this orbital pair does not present antibonding states in Figure 5b. On the other hand, the antibonding states between −4.5 and −3 eV of the Fe 4s-O 2s orbital pair of Figure 5b (CO_2_(*e*)@Fe-0N configuration) do not appear in Figure 7c,d (CO_2_(*e*)@Fe-3N configuration). This causes the contribution of this orbital pair to increase.

The ICOHP values of the most important majority and minority orbitals pair in the interaction of CO_2_ with Fe in CO_2_(*e*)@Fe-3N configuration are listed in Table 2. The contributions of the majority (ICOHP = 0.042 eV) and minority (ICOHP = 0.039 eV) orbitals between Fe and C are little affected by spin polarization. This changes in the case of the interaction between Fe and O, where the majority states (ICOHP = −0.035 eV) contribute less than the minority states (ICOHP = −0.066 eV). This is explained by the contribution of the antibonding states between −7 and −1.5 eV of the orbital pairs Fe 3d-O 2s and Fe 3d-O 2p as mentioned above (see Figure 7c). The bond strength between Fe and C (CO_2_) (ICOHP = 0.079 eV) remains unaltered as in the case of the CO_2_(*e*)@Fe-0N system. The biggest contributions to this bond are the Fe 3d-C 2p (ICOHP = 0.007 eV) and Fe 4s-C 2p (ICOHP = 0.003 eV) orbital pairs. On the other hand, the strength of the bond between Fe and O (CO_2_) (ICOHP = −0.102 eV) slightly decreases in relation to the CO_2_(*e*)@Fe-0N configuration because the weakening of the majority antibonding states of the orbital pairs Fe 3d-O 2s and Fe 3d-O 2p. The biggest contributions to this bond are the Fe 4s-C 2s (ICOHP = −0.035 eV) and Fe 4s-C 2p (ICOHP = −0.022 eV) orbital pairs. The CO_2_(*e*)@Fe-3N configuration is more stable than the CO_2_(*e*)@Fe-0N configuration because it has lower *E*_ads_. The interaction between the molecule and the iron atom is manifested in the change of position of some states in the PDOS plots close to the Fermi energy due to the spin polarization of the iron states. However, their hybridization remains low. This results in a hard charge transfer to the molecule and thus in an unaltered geometry. That is why, it could be called “strong physisorption”.

**Table 2:** Majority, Minority, and Total ICOHP Values of the Main Orbital Pairs in CO_2_(*s*)@Fe-3N and CO_2_(*s*)@Fe-3N Configuration

orbital pair (CO_2_(*e*)@Fe-3N)	majority ICOHP (eV)	minority ICOHP (eV)	total ICOHP (eV)
Fe 4s-C 2p	0.002	0.001	0.003
Fe 3d-C 2p	0.003	0.004	0.007
Fe 4s-O 2s	–0.017	–0.018	–0.035
Fe 3d-O 2p	–0.001	–0.018	–0.019
orbital pair (CO_2_(*s*)@Fe-3N)			
Fe 3d-C 2s	–0.004	–0.021	–0.025
Fe 4s-C 2s	–0.009	–0.033	–0.041
Fe 3d-C 2p	–0.025	–0.045	–0.070
Fe 3d-O 2p	0.022	–0.047	–0.025
Fe 4s-O 2s	–0.017	–0.017	–0.034
Fe 4s-O 2p	–0.033	–0.002	–0.035

The chemical interactions in the CO_2_(*s*)@Fe-3N configuration are described from the −COHP curves for the Fe–C and Fe–O bonds and are presented in Figure 7e–h. According to the figure, the peaks of the bonding and antibonding states coincide with the resonant peaks discussed above. In Figure 6e, the majority antibonding states between −4.05 and −2.88 eV correspond to the orbital pairs Fe 3d-C 2p and Fe 3d-C 2s. These antibonding states of the Fe 3d-C 2p orbital pair are not present in the CO_2_(*s*)@Fe-0N configuration (see Figure 5c) and therefore weaken their contribution in the CO_2_(*s*)@Fe-3N configuration. These antibonding states contribute considerably to the difference between the majority and minority contributions for the stability of the Fe–C bond. On the other hand, the minority states have almost no antibonding states in that energy region. There is only one antibonding state at −3.0 eV, although it is very small (see Figure 7f). In the case of the Fe–O1 bond, the Fe 3d-O 2s orbital pair is associated with antibonding states, as shown in Figure 7g,h. This orbital pair weakens the interaction between the iron atom and the oxygen atom in end-on and side-on adsorption. The Fe 3d-O 2p orbital pair has antibonding majority states at −3.94 and −0.69 eV (see Figure 7g). However, in the case of the minority states of this orbital pair, almost all are bonding states. Due to the absence of minority antibonding states, the contribution of the Fe 3d-O 2p orbital pair is higher compared to the CO_2_(*s*)@Fe-0N configuration in which antibonding states are mostly present between −4.0 eV up to the Fermi energy (see Figure 5d). Figure 7g,h shows the bonding states of the Fe 4s-O 2s orbital pair from −7.0 eV up to the Fermi energy, which differs from the case of the CO_2_(*s*)@Fe-0N configuration in this energy range (see Figure 5d). These bonding states increase the contribution of the Fe 4s-O 2s orbital pair in CO_2_(*s*)@Fe-3N. Furthermore, in the case of the Fe 4s-O 2p orbital pair, three bonding majority states are observed at −8.52, −6.84, and −2.51 eV (see Figure 7g), and there is a small region of antibonding states compared to the CO_2_(*s*)@Fe-0N configuration around −4.0 eV. A minority antibonding state is also observed at −0.44 eV in Figure 7h, which decreases the contribution of the minority states to the strengthening of the Fe–O1 bond. Although the minority states do not contribute much, the majority states compensate in such a way that the Fe 4s-O 2p orbital pair ends up contributing more to the CO_2_(*s*)@Fe-3N configuration than in the CO_2_(*s*)@Fe-0N configuration.

Table 2 lists the ICOHP values of the main orbital pairs for the CO_2_(*s*)@Fe-3N configuration. The strength of the Fe–C bond (ICOHP = −0.245 eV) in the CO_2_(*s*)@Fe-3N configuration is very similar to that in the CO_2_(*s*)@Fe-0N configuration. However, the minority states (ICOHP = −0.169 eV) contribute more to the Fe–C bond stability than the majority states (ICOHP = −0.076 eV) due to the spin polarization of the iron atom. On the other hand, the strength of the Fe–O bond (ICOHP = −0.110 eV) increased markedly in relation to the CO_2_(*s*)@Fe-0N configuration. As previously discussed, the ICOHP value of the Fe–O bond in the CO_2_(*s*)@Fe-0N configuration is −0.076 eV. The difference in Fe–O bond contribution of the majority (ICOHP = −0.044 eV) and minority (ICOHP = −0.065 eV) is caused by the spin polarization of the iron atom. The orbital pairs that most contribute to the Fe–C bond are Fe 3d-C 2p (ICOHP = −0.070 eV) and Fe 4s-C 2s (ICOHP = −0.041 eV). In the case of the Fe–O bond, the orbital pairs that most contribute are Fe 3d-O 2p (ICOHP = −0.025 eV), Fe 4s-O 2p (ICOHP = −0.035 eV), and Fe 4s-O 2s (ICOHP = −0.034 eV). It should be noted that the Fe–C and Fe–O bonds in the CO_2_(*s*)@Fe-3N system are weaker and stronger, respectively, compared to the CO_2_(*s*)@Fe-0N system.

The ICOHP curves were analyzed when using the vdW functional in the study of adsorption on Fe-3N, and the results can be seen in Figure S9. In this case, there are differences and similarities in relation to the results obtained with PBE. In the case of the end-on configuration, the greatest difference is in the Fe 3d-C 2s orbital pair (ICOHP = −0.001 eV) that presents bonding and antibonding states between −7 and −2 eV (see Figure S9a), and then the other orbital pairs do not show major differences. In the case of the side-on configuration, the most drastic change is in the Fe 3d-C 2p orbital pair (ICOHP = 0.011 eV). As noted in Table 2 and discussed above, the Fe 3d-C 2p orbital pair is a bonding state with the PBE. However, it manifests as an antibonding orbital pair with the vdW.

### Evolution of Adsorption Characteristics Change of Pyridinic-N Coordination

**Figure 8:**
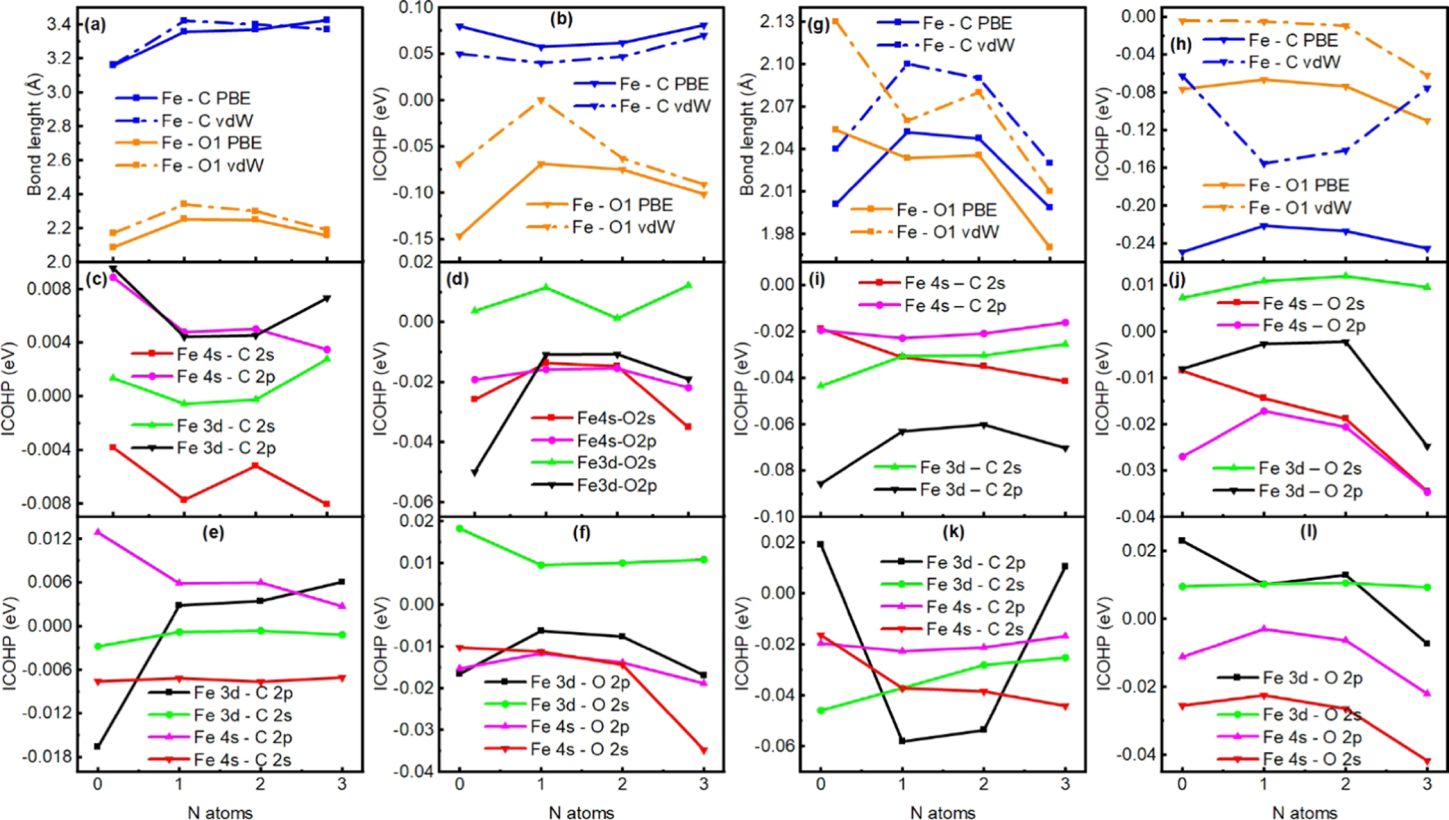
Relationship between CO_2_–Fe distances for PBE and vdW methods (a), total Fe–C and Fe–O1 ICOHP value (b), ICOHP values per orbital pair of Fe–C (c), and Fe–O (d), ICOHP values for vdW, per orbital pair of Fe–C (e), and Fe–O (f) bonds with pyridinic-N coordination in end-on adsorption. Relationship between CO_2_–Fe distances (g), total Fe–O1 ICOHP values (h), ICOHP values per orbital pair of Fe–C (i) and Fe–O (j), for vdW the orbital pair of Fe–C and Fe–O1 are in (k) and (l) bonds with pyridinic-N coordination in side-on adsorption.

To better understand the relationship between the coordination and chemical interactions, the distances between the iron atom and the molecule are compared to the ICOHP values in relation to pyridinic-N coordination. As shown in Figure 8a,b, the Fe–O1 distance and its ICOHP values follow the same trend in end-on adsorption. However, the distance slightly increases by 0.1 Å even when *E*_ads_ decreases, which seems to be contradictory, but it is explained by considering the changes in chemical interactions. Figure 8 also shows the evolution with the changes in the coordination of the ICOHP values of the main orbital pairs in the end-on adsorption of CO_2_. Figure 8c,d shows the contributions for the Fe–C and Fe–O bonds, respectively, as the coordination changes. As mentioned above, the states related to the Fe–C bond are mostly antibonding (Figure 8c). In the case of the Fe–O bond, the most important orbital pair is Fe 3d-O 2p in the CO_2_(*e*)@Fe-0N configuration. However, this changes with the increase in pyridinic-N coordination to such an extent that the Fe 4s-O 2s and Fe 4s-O 2p orbital pairs become more important, surpassing Fe 3d-O 2p (Figure 8d). This indicates that the structure becomes more stable with the sigma nature of the bond, related to the interaction between s orbitals, caused by the pyridinic-N coordination increment. This is the reason for the inclination of the CO_2_ molecule with the *z* axis, disfavoring the π-bonding nature. This change in the nature of the bonding between iron and oxygen makes the adsorption more stable. However, the Fe–O1 bond length does not change monotonically.

Figure 8e,f shows the bond lengths and ICOHP values for side-on adsorption. It can be clearly seen from Figure 8e that there is a reversal of the bonds as the pyridinic-N coordination changes. The carbon atom is closer to the iron atom than the oxygen atom in the CO_2_(*s*)@Fe-0N configuration. However, the oxygen atom approaches the iron atom in such a way that it becomes closer to the iron atom than to the carbon atom as the pyridinic-N coordination increases. The ICOHP values follow the trend of the changes in the length of the bonds (see Figure 8f). Figure 8g,h indicates that as the coordination is modified, there is a strengthening of the sigma nature of the bonds related to the interaction between s orbitals (Fe 4s-C 2s and Fe 4s-O 2s) since they do not have directional preference. However, the interaction between Fe 3d and O 2p orbitals is weakened by the asymmetry generated by the doping of a nitrogen atom. As the symmetry is restored by increasing pyridinic-N coordination, the interaction related to the Fe 3d and O 2p orbitals becomes stronger.

Figure 8 also shows the evolution of the structural parameters and chemical interactions with modification of the coordination with the vdW functional. In the case of the end-on configuration, the bonds and chemical interaction between the Fe and C atoms vary slightly, as seen in Figure 8a,b. The most important differences are the chemical interactions between Fe and O1 when the adsorption occurs on the Fe-0N and Fe-1N substrates (see Figure 8b). This difference is due to the Fe 3d-O 2p orbital pair, which has a different behavior than that described by PBE, as can be seen in Figure 8d,f. Also contributing is the fact that the other orbital pairs reduce their interaction by almost half until in Fe-3N, the Fe 4s-O 2s orbital pair intensifies. In the case of the side-on configuration, the Fe 3d-C 2p orbital pair dominates in a certain way the chemical interaction between the Fe and C atom, where it can be seen that the curve of the mentioned orbital pair (Figure 8k) has a behavior very similar to that of Figure 8h. However, the interaction between the Fe atom and O1 is dominated by the Fe 4s-O 2s pair orbital for both PBE and vdW. In summary, the most important effect of including the nonlocal correction of the vdW functional is in the description of the delocalized pi bonds.

## Conclusions

The adsorption of the CO_2_ molecule on Fe and N codoped graphene was studied considering the effect of changing the pyridinic-N coordination of Fe and the orientation, side-on and end-on. It was found that the interaction between CO_2_ and the substrate improves with an increase in pyridinic-N coordination. However, the adsorption characteristics change depending on the orientation of the molecule with respect to the substrate. It was observed that the interaction is weak if CO_2_ is the end-on orientation with the graphene sheet, although the interaction increases with coordination. The adsorption becomes more energetically favorable due to the strengthening of the interaction between the s orbitals of iron and oxygen that causes the presence of nitrogen atoms. However, although the analysis of the density of states shows hybridization between the substrate and molecule orbitals and there is charge transferred to the molecule, this is not accentuated in the end-on configuration. Even the changes in the geometry of the molecule are also small. This is an indicator of physisorption. When long-range interactions are included, differences are observed with respect to what happens when using a GGA-PBE functional. The adsorption energy is almost unchanged with pyridinic coordination up to Fe-2N. This is mainly because chemical interactions are reduced.

On the other hand, side-on orientation favors adsorption. In this case, the iron atom interacts with the carbon atom of the molecule and one oxygen atom. The interaction with the two atoms improves the adsorption. Changes in coordination not only produce an increase in the interaction between s orbitals as in the case of end-on adsorption but also intensify the interaction of the 3d orbitals of iron and the 2p orbitals of the molecule. A strong hybridization between the orbitals of iron and the molecule is observed at the density of states near the Fermi energy, and the charge transferred to the molecule is almost an order of magnitude greater than in the case of the end-on configuration. The above is accompanied by a drastic change in the structure. Due to this, we can affirm that the molecule is activated in the chemisorption process. As in the case of the end-on configuration, the chemical interactions are reduced when the vdW functional is included until they are intensified when the three nitrogen atoms are present in the substrate structure. The results obtained show that the coordination change modifies the adsorption characteristics of the molecule until chemisorption by a graphene-based adsorbent. This can be useful to regulate the activation of CO_2_ in a catalytic process in this type of adsorbent.

## Supporting Information Available

Adsorption energy, geometric parameters, charge transfer to the iron atom and magnetic moment, PDOS for Fe-0N, Fe-1N, Fe-2N, and Fe-3N, initial side-on configurations for CO_2_(*s*)@Fe-0N and total density of states of the CO_2_ molecule; methodology for the calculation of BSSE; BSSE calculated parameters (Table S1); comparison between 4 × 4 and 5 × 5 supercells (Table S2); main physical characteristics of the substrates (Table S3); PDOS of the substrates (Figure S1); PDOS of Fe-0N and Fe-3N substrates using GGA+*U* (Figure S2); initial configurations of the CO_2_(*s*)@Fe-0N system (Figure S3); molecular dynamics simulation of CO_2_(*s*)@Fe-0N system at 500 K (Figure S4); total density of states of the CO2 molecule (Figure S5); PDOS of CO_2_ and Fe atom on Fe-0N with vdW (Figure S6); COHP curves in the CO_2_(*e*)@Fe-0N with vdW (Figure S7); PDOS of CO_2_ and Fe atom on Fe-3N with vdW (Figure S8); and COHP curves in the CO_2_(*s*)@Fe-0N with vdW (Figure S9) (PDF)
Ab initio molecular dynamics simulation of the transition from end-on to side-on configuration of the CO_2_ molecule adsorbed on Fe-0N substrate at 1000 K (AVI)

## Supplementary Material

la3c03327_si_001.pdf

la3c03327_si_002.avi
