# Supplementary material for: Pyridinic-N Coordination Effect on the Adsorption and Activation of CO2 by Single Vacancy Iron-Doped Graphene
Source: Langmuir. 2024 Mar 18;40(13):6703–17. doi: 10.1021/acs.langmuir.3c03327 (PMC10993407; doi:10.1021/acs.langmuir.3c03327)
Supplement: Supplementary file 1 — la3c03327_si_001.pdf [file la3c03327_si_001.pdf]

Pyridinic-N coordination effect on adsorption and activation of CO_2 by single vacancy iron-doped graphene

Hugo Cabrera-Tinoco[a(*)], Luis Borja-Castro[b], Renato Valencia-Bedregal[b], Adela Perez-Carreño[a], Aldo Lalupu-García[a], Ismael Veliz-Quiñones[a], Angel Guillermo Bustamante Dominguez[b], Crispin H.W. Barnes[c], Luis De Los Santos Valladares[c,d(*)]

[a] Área de Ciencias Básicas, Universidad Continental, Lima 15311, Perú
[b] Laboratorio de Cerámicos y Nanomateriales, Facultad de Ciencias Físicas, Universidad Nacional Mayor de San Marcos, Ap. Postal 14-0149, Lima, Perú
[c] Programa de Pós-Graduação em Ciências de Materiais, Centro de Ciências Exatas e da Natureza, Universidade Federal de Pernambuco, 50670-901 Recife-PE, Brazil
[d] Cavendish Laboratory, Department of Physics, University of Cambridge, J. J Thomson Av, Cambridge CB3 0H3, UK
(*) Corresponding Authors emails: hcabrera@continental.edu.pe and ld301@cam.ac.uk

Supporting Information

Table of contents

Methodology for the calculation of BSSE
Table S1 with the BSSE calculated parameters
Table S2 with the comparison between 4x4 and 5x5 supercells
Main physical characteristics of the substrates (Table S3)
PDOS of the substrates (Figure S1)
PDOS of Fe-0N and Fe-3N substrates using GGA+U (Figure S2)
Initial configurations of the CO_2(s)@Fe-0N system (Figure S3)
Molecular dynamics simulation of CO_2(s)@Fe-0N system at 500 K (Figure S4)
Total density of states of the CO2 molecule (Figure S5)
PDOS of CO_2 and Fe atom on Fe-0N with vdW (Figure S6)
COHP curves in the CO_2(e)@Fe-0N with vdW (Figure S7)
PDOS of CO_2 and Fe atom on Fe-3N with vdW (Figure S8)
COHP curves in the CO_2(s)@Fe-0N with vdW (Figure S9)

S1.1 Metodology for basis set superposition error calculation

To calculate the error produced by BSSE we use the counterpoise correction. To do this, we are going to define two systems that we will call A and B. A in our case is the substrate (Fe-0N, Fe-1N, Fe-2N and Fe-3N) and B is the molecule (CO_2). We define the notation of the energies in the following way: in the superscript the basis set used is indicated, in the subscript the geometry is indicated and in parentheses the system considered.

For example, the term $E_{AB}^{AB}(A)$ is the energy corresponding to the calculation of system B (CO_2 only) with the geometry it has when it is activated and using its own basis functions together with the "ghosts" basis functions of the substrate. They are called ghost basis functions because they are functions of system A (the substrate in our example) that are added to the functions of system B (CO_2

in our example) to perform the single point calculation but without modifying the number of electrons, that is, they are “empty” functions basis. Then the BSSE-corrected adsorption energy is given by

$$E_{ads}^{corrected} = [E_{AB}^{AB}(AB) - E_{AB}^{AB}(A) - E_{AB}^{AB}(B)] + [E_{AB}^{A}(A) - E_{A}^{A}(A)] + [E_{AB}^{B}(B) - E_{B}^{B}(B)] \quad (S1).$$

The following table shows the energies (eV) established in the equation for the CO_2(e)@Fe-0N and CO_2(s)@Fe-3N systems.

Table S1. Values of E_{AB}^{AB}(AB), E_{AB}^{AB}(A), E_{AB}^{AB}(B), E_{AB}^{A}(A), E_{A}^{A}(A), E_{AB}^{B}(B), E_{B}^{B}(B) and E_{ads}^{corr}(A) of CO_2(e)@Fe-0N and CO_2(s)@Fe-3N configurations.

Configuration	E_{AB}^{AB}(AB)	E_{AB}^{AB}(A)	E_{AB}^{AB}(B)	E_{AB}^{A}(A)	E_{A}^{A}(A)	E_{AB}^{B}(B)	E_{B}^{B}(B)	E_{ads}^{corr}
CO_2(s) @Fe-3N	-6819.073734	-5765.551155	-1048.520563	-5765.446545	-5766.476083	-1048.461464	-1049.889877	-2.544065
CO_2(e) @Fe-0N	-6441.362420	-5391.079827	-1049.970093	-5391.055401	-5391.060375	-1049.878980	-1049.889877	−0.296629

Table S2. Comparison of binding energy (eV), transferred charge (e) and geometric characteristics between the 4x4 and 5x5 supercell in the end-on configuration

Substrate	E_{bin} (eV)	d(C-O1)	d(C-O2)	d(Fe-O1)	d(Fe-C)	d(Fe-N)	θ(CO2)	θ(Fe-CO2)	Δq(CO2)	Δq(Fe)
Fe-0N 4x4	-0.412	1.198	1.183	2.088	1.78<br>1.78<br>1.78		179.844	146.44	-0.03	0.946
Fe-0N 5x5	-0.426	1.198	1.182	2.092	1.79<br>1.79<br>1.79		179.640	147.83	-0.03	1.00
Fe-3N 4x4	-1.962	1.196	1.187	2.16	-	1.96<br>1.96<br>1.94	179.735	164.64	-0.06	1.21
Fe-3N 5x5	-2.017	1.194	1.183	2.19	-	1.96<br>1.96<br>1.94	179.735	159.80	-0.04	1.26

Table S3. binding energy, geometric parameters, charge transfer to the iron atom and magnetic moment.

Substrate	E_{bin} (eV)	$d_{Fe\text{-}C}$ (Å)	$d_{Fe\text{-}N}$ (Å)	h (Å)	Δq_{Fe} (e)	M(μ_B)
Fe-0N	-5.847573	1.7794	-	1.40	0.732096	0.000001
Fe-1N	-4.033924	1.8001	1.8098	1.38	0.875335	1.000203
Fe-2N	-4.104342	1.7875	1.8038	1.34	0.901204	0.000001
Fe-3N	-3.023631	-	1.8069	1.28	0.98376	-0.000002

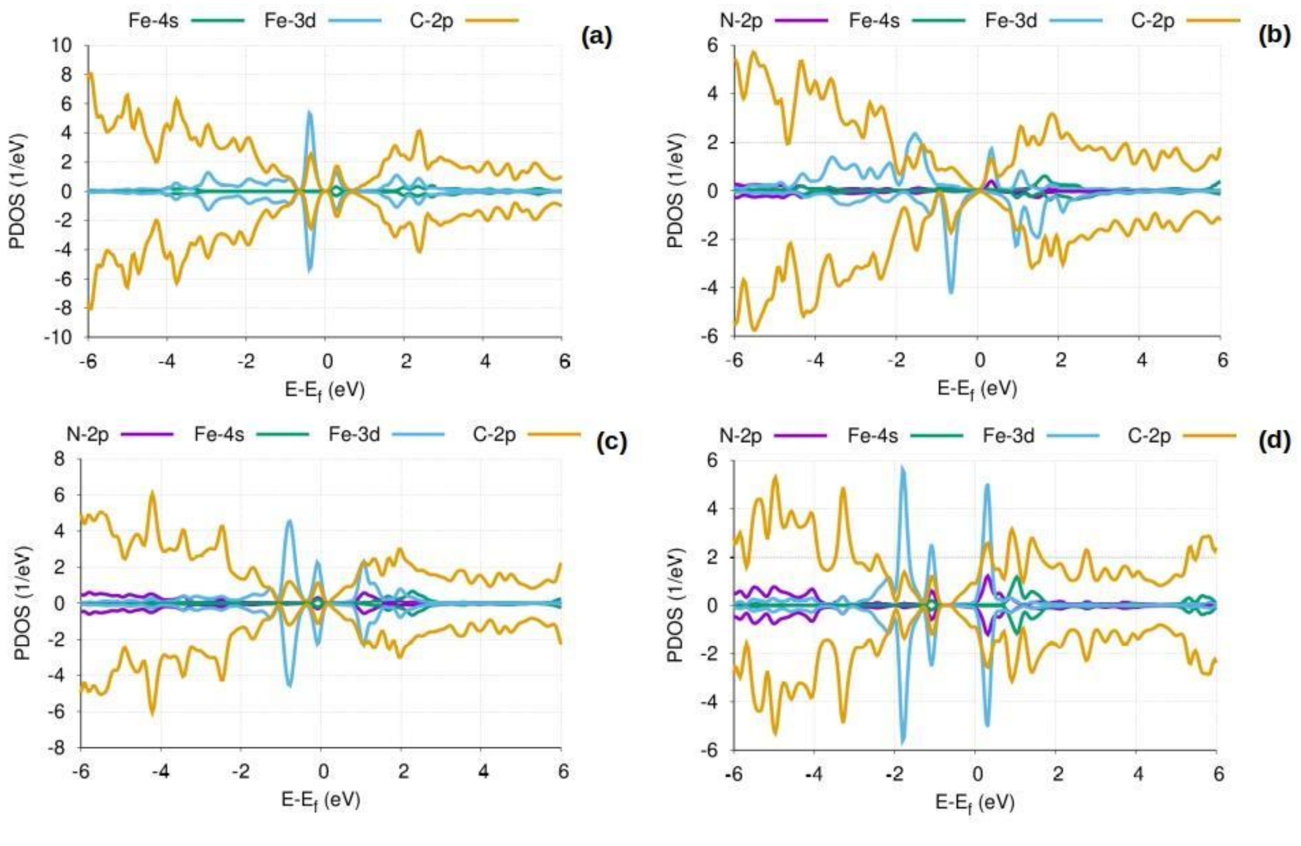

Figure S1. PDOS of Fe-0N (a), Fe-1N (b), Fe-2N (c) and Fe-3N (d) substrate.

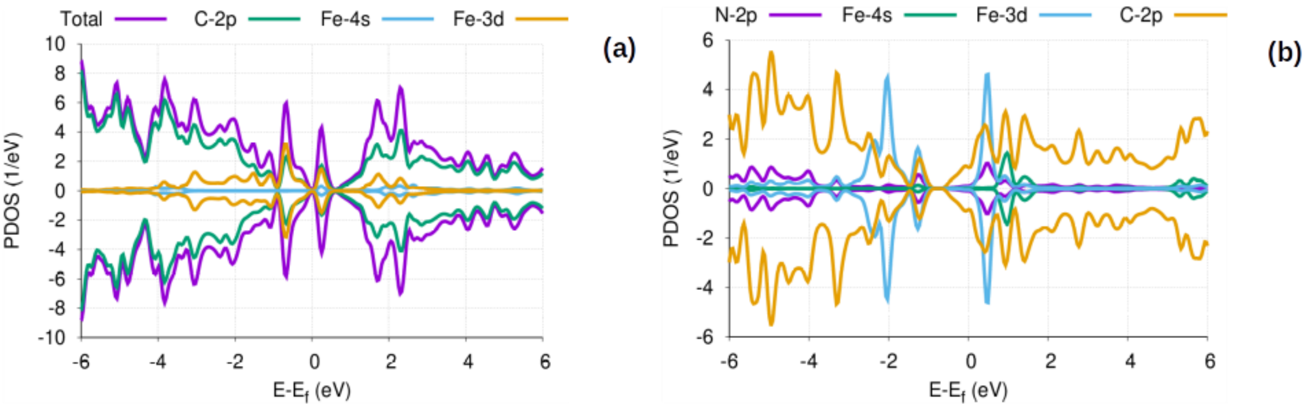

Figure S2. PDOS of the Fe-0N (a) and Fe-3N (b) substrate using GGA+U with U-J=1 eV.

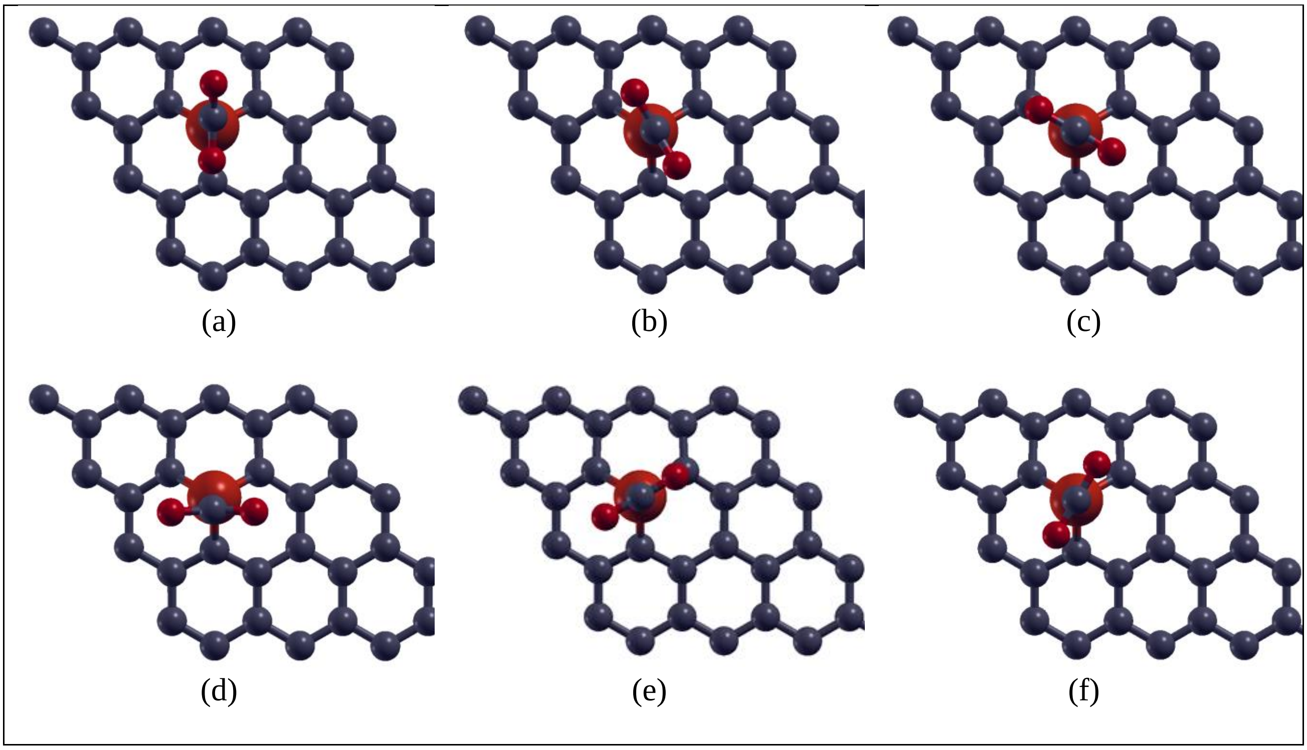

Figure S3. Initial horizontal configurations for CO_2(s) @Fe-0N. The CO_2 molecule was rotated in such a way that the configurations are 0° (a), 30° (b), 60° (c), 90° (d), 120° (e) and 150° (f) degrees relative to the carbon atom below the iron atom on the y-axis. These same structures were used as initial configurations with pyridinic-N coordination.

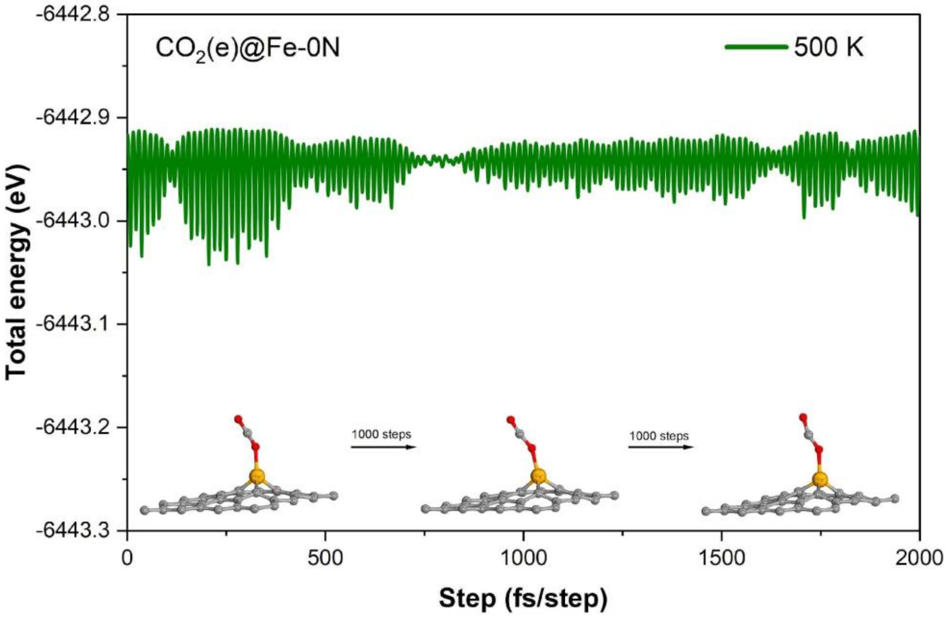

Figure S4. Ab-initio Molecular dynamics of CO_2(e)@Fe-0N configuration at 500 K.

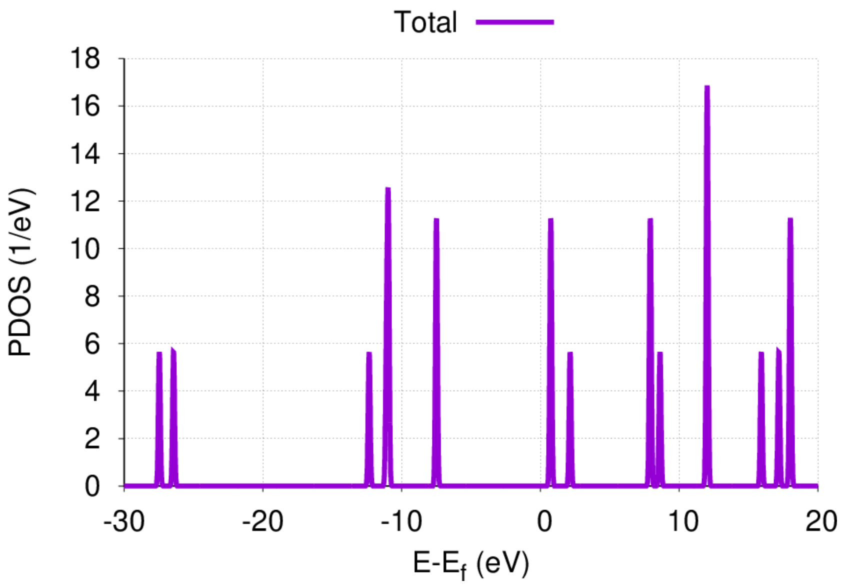

Figure S5. Total density of states of the CO_2 molecule.

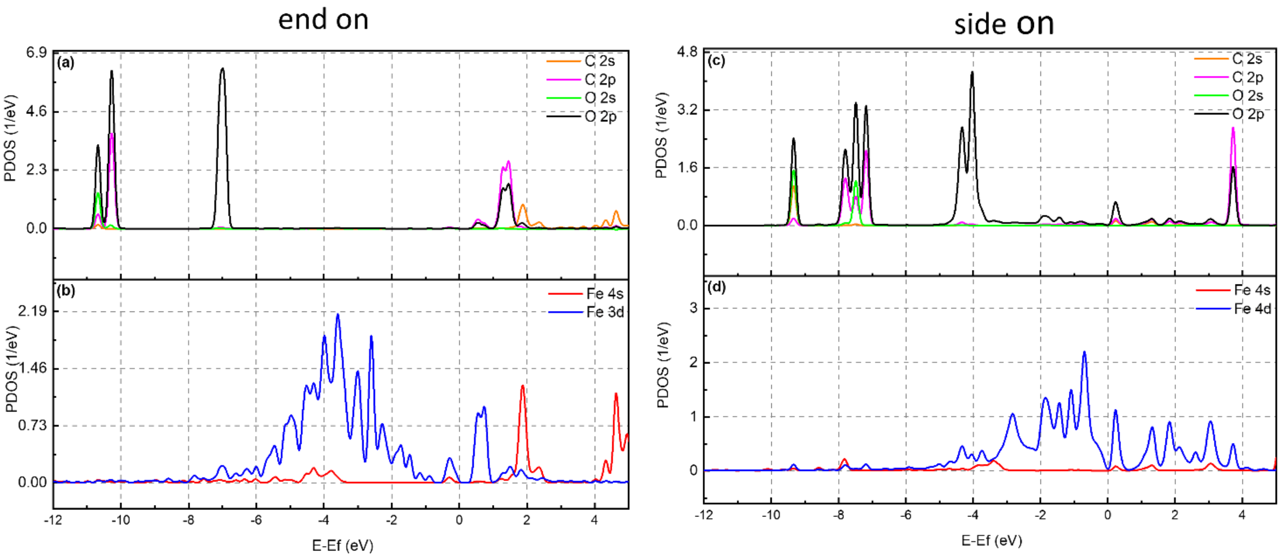

Figure S6. PDOS curves of CO_2 (a) and the Fe atom (b) in the CO_2(e)@Fe-0N configuration with vdW. PDOS curves of CO_2 (c) and the Fe atom (d) in the CO_2(s)@Fe-0N configuration with vdW.

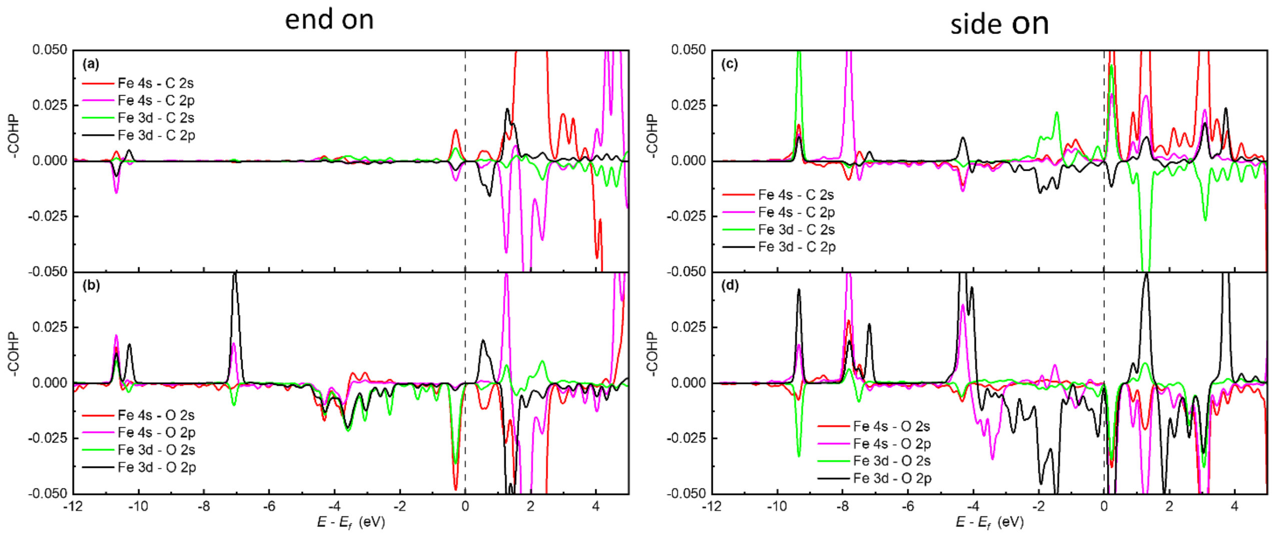

Figure S7. COHP curves of Fe-C (CO_2) (a) and Fe-O (b) orbital-pairs in CO_2(e)@Fe-0N configuration with vdW. COHP curves of Fe-C (CO_2) (c) and Fe-O (d) orbital-pairs in CO_2(s)@Fe-0N configuration with vdW.

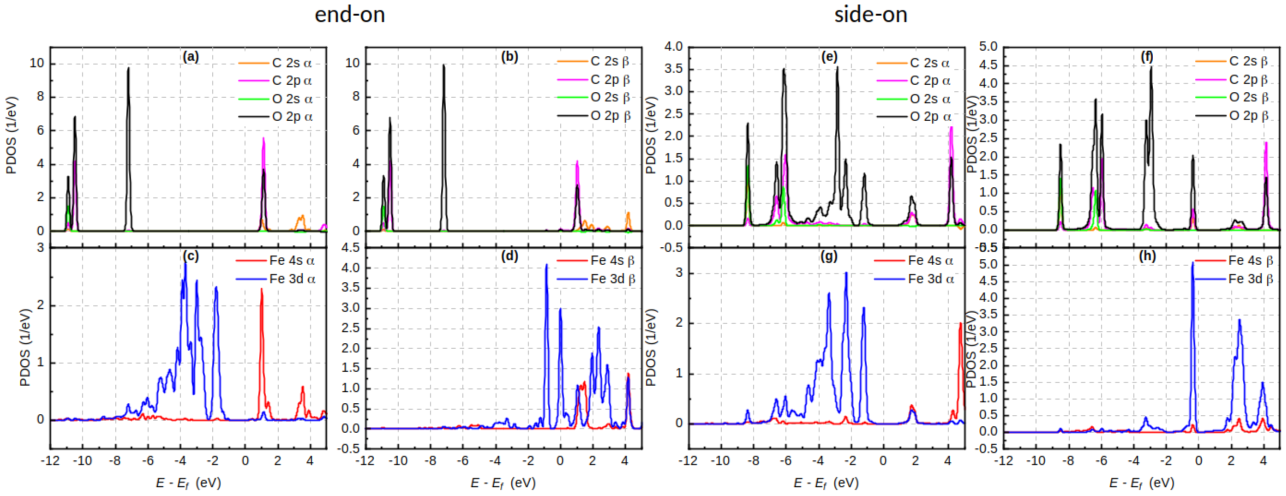

Figure S8. PDOS curves of CO_2 majority (a), minority (b), Fe atom majority (c) and minority (d) for CO_2(e)@Fe-3N configuration with vdW. PDOS curves of CO_2 majority (e), minority (f), Fe atom majority (g) and minority (h) for CO_2(s)@Fe-3N configuration with vdW.

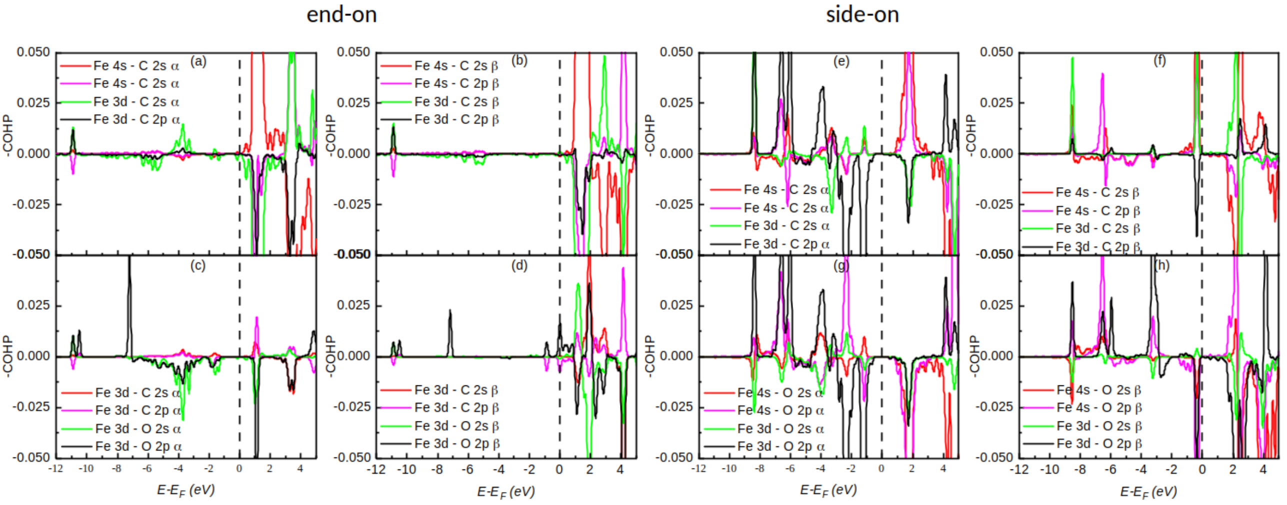

Figure S9. COHP curves of majority Fe-C (CO_2) (a), minority Fe-C (CO_2) (b), majority Fe-O (c) and minority Fe-O (d) orbital-pairs in CO_2(e)@Fe-3N configuration with vdW. COHP curves of majority Fe-C (CO_2) (e), minority Fe-C (CO_2) (f), majority Fe-O (g) and minority Fe-O (h) in CO_2(s)@Fe-3N configuration with vdW.
